# Inhibitory to non-inhibitory evolution of the *ζ* subunit of the F_1_F_O_-ATPase of *Paracoccus denitrificans* and *α*-proteobacteria as related to mitochondrial endosymbiosis

**DOI:** 10.3389/fmolb.2023.1184200

**Published:** 2023-08-17

**Authors:** Francisco Mendoza-Hoffmann, Lingyun Yang, Damiano Buratto, Jorge Brito-Sánchez, Gilberto Garduño-Javier, Emiliano Salinas-López, Cristina Uribe-Álvarez, Raquel Ortega, Oliver Sotelo-Serrano, Miguel Ángel Cevallos, Leticia Ramírez-Silva, Salvador Uribe-Carvajal, Gerardo Pérez-Hernández, Heliodoro Celis-Sandoval, José J. García-Trejo

**Affiliations:** ^1^ Departamento de Biología, Facultad de Química, Ciudad Universitaria, Universidad Nacional Autónoma de México (U.N.A.M.), Ciudad de México, México; ^2^ iHuman Institute, ShanghaiTech University, Shanghai, China; ^3^ Shanghai Institute for Advanced Immunochemical Studies, ShanghaiTech University, Shanghai, China; ^4^ Instituto de Fisiología Celular, Universidad Nacional Autónoma de México (U.N.A.M.), Ciudad de México, México; ^5^ Centro de Ciencias Genómicas, Universidad Nacional Autónoma de México (U.N.A.M.), Ciudad de México, México; ^6^ Departamento de Bioquímica, Facultad de Medicina, Universidad Nacional Autónoma de México (U.N.A.M.), Ciudad de México, México; ^7^ Departamento de Ciencias Naturales, Universidad Autónoma Metropolitana, Unidad Cuajimalpa, Ciudad de México, México

**Keywords:** evolution, ATP synthase, α-proteobacteria, mitochondria, endosymbiosis, zeta subunit, ζ, *Paracoccus denitrificans*

## Abstract

**Introduction:** The *ζ* subunit is a potent inhibitor of the F_1_F_O_-ATPase of *Paracoccus denitrificans* (PdF_1_F_O_-ATPase) and related *α*-proteobacteria different from the other two canonical inhibitors of bacterial (*ε*) and mitochondrial (IF_1_) F_1_F_O_-ATPases. *ζ* mimics mitochondrial IF_1_ in its inhibitory N-terminus, blocking the PdF_1_F_O_-ATPase activity as a unidirectional pawl-ratchet and allowing the PdF_1_F_O_-ATP synthase turnover. *ζ* is essential for the respiratory growth of *P. denitrificans*, as we showed by a *Δζ* knockout. Given the vital role of ζ in the physiology of *P. denitrificans*, here, we assessed the evolution of *ζ* across the *α*-proteobacteria class.

**Methods:** Through bioinformatic, biochemical, molecular biology, functional, and structural analyses of several *ζ* subunits, we confirmed the conservation of the inhibitory N-terminus of *ζ* and its divergence toward its C-terminus. We reconstituted homologously or heterologously the recombinant *ζ* subunits from several *α*-proteobacteria into the respective F-ATPases, including free-living photosynthetic, facultative symbiont, and intracellular facultative or obligate parasitic α-proteobacteria.

**Results and discussion:** The results show that *ζ* evolved, preserving its inhibitory function in free-living α-proteobacteria exposed to broad environmental changes that could compromise the cellular ATP pools. However, the *ζ* inhibitory function was diminished or lost in some symbiotic α-proteobacteria where *ζ* is non-essential given the possible exchange of nutrients and ATP from hosts. Accordingly, the *ζ* gene is absent in some strictly parasitic pathogenic Rickettsiales, which may obtain ATP from the parasitized hosts. We also resolved the NMR structure of the *ζ* subunit of *Sinorhizobium meliloti* (Sm-*ζ*) and compared it with its structure modeled in AlphaFold. We found a transition from a compact ordered non-inhibitory conformation into an extended α-helical inhibitory N-terminus conformation, thus explaining why the Sm-*ζ* cannot exert homologous inhibition. However, it is still able to inhibit the PdF_1_F_O_-ATPase heterologously. Together with the loss of the inhibitory function of α-proteobacterial *ε*, the data confirm that the primary inhibitory function of the α-proteobacterial F_1_F_O_-ATPase was transferred from *ε* to *ζ* and that ζ, ε, and IF_1_ evolved by convergent evolution. Some key evolutionary implications on the endosymbiotic origin of mitochondria, as most likely derived from *α*-proteobacteria, are also discussed.

## Introduction

The F_1_F_O_-ATP synthase is the ubiquitous nanomotor that fuels life with the chemical energy of ATP to drive most of the endergonic biochemical reactions and work processes in cells. The ATP synthase is the terminal multiprotein complex of oxidative phosphorylation or photophosphorylation of most living beings. When the transmembrane ion gradient is partially or totally collapsed (in ischemia, anaerobiosis, uncoupling in bacteria and mitochondria, or in the darkness in photophosphorylation), the ATP synthase is thermodynamically prone to reverse its intrinsic rotation from clockwise (CW, viewed from F_O_ to F_1_) to counterclockwise (CCW) and, therefore, to hydrolyze ATP, thus becoming an ATP-driven primary ion pump. To prevent the futile and deleterious F_1_F_O_-ATPase activity, the ATP synthases of different organisms have acquired different inhibitory proteins or protein domains. Thus, nature designed, by evolution, several F_1_F_O_-ATPase inhibitory proteins, such as bacterial ε (Sternweis and Smith, 1980), *α*-proteobacterial *ζ* (de la Rosa-Morales, 2005; Morales-Rios et al., 2010), and mitochondrial IF_1_ (Pullman and Monroy, 1963), to fully prevent futile ATP hydrolysis (Krah, 2015; Mendoza-Hoffmann et al., 2018; Zarco-Zavala et al., 2018). These proteins complement a partial MgADP inhibitory mechanism present in ATP synthases, including the one of *Paracoccus denitrificans* (Zharova and Vinogradov, 2004), which has been demonstrated *in vitro* but not *in vivo.* Therefore*,* in the case that it actually exists *in vivo*, this Mg-ADP inhibition is evidently insufficient to prevent wasteful F_1_F_O_-ATPase activity. Otherwise, the inhibitory functions of bacterial *ε*, *α*-proteobacterial *ζ*, and mitochondrial IF_1_ would be physiologically and evolutionarily unnecessary. Recently, we showed that this inhibitory MgADP only partially inhibits the F_1_F_O_-ATPase activity in *Paracoccus denitrificans* (PdF_1_F_O_-ATPase) (Zarco-Zavala et al., 2020). In contrast, the natural inhibitory “zeta” (*ζ*) subunit of this PdF_1_F_O_-ATPase (Morales-Rios et al., 2010; Zarco-Zavala et al., 2014; Garcia-Trejo et al., 2016) exerts a full inhibitory activity that completely stalls the F_1_-ATPase (Zarco-Zavala et al., 2020). The key biological role of *ζ* has also been illustrated by the severe delay in respiratory growth of a *ζ* null *P. denitrificans* knockout mutant relative to the wild-type strain (Pd1222) (Mendoza-Hoffmann et al., 2018a, 2018b). This Pd*Δζ* mutant also demonstrated the unidirectional pawl/ratchet mechanism of *ζ* to block the reverse PdF_1_F_O_-ATPase activity selectively and allow the full PdF_1_F_O_-ATP synthase turnover rate to favor the cellular bioenergetic ATP production (Mendoza-Hoffmann et al., 2018a, 2018b). This Pd*Δζ* mutant also supported that the same key role and unidirectional pawl/ratchet mechanism of *ζ* very likely also works for the other inhibitory bacterial ε and mitochondrial IF_1_ proteins, although with different structures (Mendoza-Hoffmann et al., 2018a, 2018b; Zarco-Zavala et al., 2018). Recent studies by other researchers suggest that besides MgADP and *ζ*, the ε subunit has an apparent inhibitory role in *P. denitrificans* (Pd-*ε*). However, instead of showing a clear activation in their truncated Pd-*ε* ^ΔCT^ mutants, their results show that the stronger and more significant PdF_1_F_O_-ATPase activation occurred exclusively when ζ was deleted but not when only Pd-*ε* was truncated (Varghese et al., 2018; Jarman et al., 2021); the details of these studies are discussed in Supplementary Material. It is evident that, in general, the *ε* subunit from *P. denitrificans* and most, if not all, *α*-proteobacteria have completely lost the inhibitory and ATP binding properties present in other non-α-proteobacterial inhibitory ε subunits (see the work of Zarco-Zavala et al. (2014), Mendoza-Hoffmann et al. (2018a, 2018b), and Mendoza-Hoffmann et al. (2022) and Supplementary Material); therefore, the *ζ* subunit acquired fully these inhibitory and regulatory properties in *P. denitrificans* and related *α*-proteobacteria.

Given its biological relevance, we studied here the evolution of *ζ* across the *α*-proteobacteria class. To this aim, we carried out bioinformatic, molecular biology, biochemical, and structural analyses of several *ζ* subunits in distinct bacterial families, including strictly free-living respiratory (*Paracoccus denitrificans*, Pd-*ζ*) or photosynthetic (*Cereibacter sphaeroides,* Cs-ζ, *and Rhodospirillum rubrum,* Rr-*ζ*), facultative symbiotic (*Rhizobium etli*, Re-*ζ*, and *Sinorhizobium meliloti*, Sm-*ζ*), and strictly parasitic (*Wolbachia pipientis,* Wp-*ζ*) α-proteobacteria. The results confirmed that the *ζ* subunit is a protein family (DUF 1476) essentially exclusive of the α-proteobacteria class. We confirmed the strong conservation of the functional N-terminal inhibitory domain of *ζ* and its divergence toward the C-terminus. We also carried out homologous or heterologous reconstitution of several *ζ* subunits into the respective α-proteobacterial F_1_ or F_1_F_O_-ATPases. The results showed that the evolution of *ζ* and the preservation or loss of its inhibitory function is in concordance with its bioenergetic requirement in different bacterial families, environments, and lifestyles. Finally, we resolved and correlated the NMR and AlphaFold (AF) structures of the *ζ* subunit from *S. meliloti* with its lack of inhibitory function. Taken together, the data show that bacterial ε, α-proteobacterial *ζ*, and mitochondrial IF_1_ emerged independently by convergent evolution as F_1_F_O_-ATPase inhibitors. These results also have important implications in the endosymbiotic evolution from α-proteobacteria to mitochondria and putative important future applications.

## Materials and methods

### Experimental methods

#### Purification of F_1_ and solubilization of F_1_F_O_

Sub-bacterial particles (SBP) from *P. denitrificans* and the other non-photosynthetic α-proteobacteria were prepared as previously described (Morales-Rios et al., 2010), and chromatophores were prepared from *R. capsulatus*, *R. rubrum,* or *C. sphaeroides* cultures, as described previously (Behrens and De Meis, 1985). The F_1_ from *Paracoccus denitrificans* Pd1222 (Pd), *Rhizobium etli* CFN42 (Re), or *Sinorhizobium meliloti* 1,023 (Sm) strains was purified from SBP or inverted membranes, as described previously (Zarco-Zavala et al., 2014). The same procedure was used to purify the F_1_-ATPase of *Rhodobacter capsulatus* from chromatophores. Solubilization of the F_1_F_O_-ATPase from SBP or chromatophores was carried out as described for the preparation of Blue Native Electrophoresis (BN-PAGE) with 2–4 mg/mg of protein of digitonin (Schagger and von Jagow, 1991).

#### F_1_ – ATPase and F_1_ F_O_ – ATPase activities

We measured the *P. denitrificans*, *R. etli*, and *S. meliloti* F_1_-ATPase activities using a pyruvate kinase and lactate dehydrogenase coupled assay that follows the NADH oxidation, as described previously (Morales-Rios et al., 2010; Zarco-Zavala et al., 2014). The reaction mixture contained 50 mM of Tris/acetate (pH 8.0), 250 mM of sucrose, 3 mM of Mg^+2^ acetate, 30 mM of K^+1^ acetate, 1.5 mM of PEP, 3 mM of ATP, 200 μM of NADH, 4 U/ml of PK, and 4 U/ml of LDH. Reactions started by adding the F_1_ (or F_1_F_O_) ATPases to the reaction cells. Measurements were carried out using 0.15% LDAO as an ATPase activator. In the ATPase assays of chromatophores or SBP, we also added 0.03 μg/μL of rotenone and 5 mM of sodium cyanide. In chromatophores, the ATPase assays also included sulfite (≤2 mM) and 2 μM of FCCP.

#### Cloning, expression, and purification of recombinant *ζ* subunits from several *α*-proteobacteria

The recombinant *ζ* subunits were PCR-amplified using genomic DNA of the strains *P. denitrificans* Pd1222, *R. etli* CFN42, *S. meliloti* 1,021, and *Cereibacter sphaeroides* 2.4.1 (formerly *Rhodobacter sphaeroides*) and designed primers (Supplementary Table S1). The ζ gene from *Rhodospirillum rubrum* ATCC 11170 inserted into pET3a was purchased from GeneScript. The ζ amplicons were ligated into a pJET1.2 subcloning vector (Thermo Fisher Scientific), and competent cells of *E. coli* DH5α were transformed with this construction. The cloned ζ genes were confirmed by NdeI and BamHI double digestion and subsequently ligated into a pT7-7 plasmid, with the exception of ζ from *C. sphaeroides* (Cs-ζ), which was cloned into pET3a (Supplementary Figure S3). The pT7-7/ζ or pET3a/ζ constructs were sequenced and then transformed into competent *E. coli* BL21 (DE3) pLys S codon plus cells. The recombinant ζ subunits were overexpressed and purified as described previously (Morales-Rios et al., 2010; Zarco-Zavala et al., 2014).

#### Circular dichroism experiments

Far UV CD measurements of Pd-ζ and Sm-ζ were carried out at a concentration of 0.07 mg/ml at 25°C on a Jasco J715 spectropolarimeter using a quartz cell with 0.1 cm pathlength. The protein samples were previously filtered and diluted in 25 mM of phosphate buffer with pH 8.0. The results were expressed as mean residue ellipticity at the wavelength (λ) given by

$$MRE=MRW\;X\;\theta_{\lambda }/\;10\;X\;d\;X\;c,$$

where MRW is the mean residue weight for the peptide bond, *θ*_λ_ is the observed ellipticity (degrees) at wavelength *λ*, d is the pathlength (cm), and c is the protein concentration (mg/ml). The spectra obtained were an average of three scans. The secondary structure content was calculated with the BeStSel online software (https://bestsel.elte.hu/index.php). See references in the link and also the work of Micsonai et al. (2022a) and Micsonai et al. (2022b) for further details.

#### Inhibitory homologous and heterologous *ζ* reconstitution assays

The F_1_, F_1_F_O_, SBP, or chromatophores from each strain (*P. denitrificans*, *R. etli*, *S. meliloti*, *R. capsulatus*, *C. sphaeroides*, or *R. rubrum*) were preincubated with the indicated concentrations of the recombinant Pd-ζ, Re-ζ, Sm-ζ, Cs-ζ, or Rr-ζ in the presence of 1 mM of sulfite and 1 mM of ATP and MgCl_2_ in a buffer containing 20 mM of Tris/HCl and 250 mM of sucrose at pH 8.0. The reconstituted samples were incubated for 20 min at room temperature; the ATPase activities of the samples were then measured by the coupled spectrophotometric method described previously.

#### ^13^C, ^15^N uniform double labeling of recombinant Sm-ζ

The ^13^C, ^15^N uniformly double-labeled Sm*-*ζ was overexpressed in *E. coli* BL21 cells transformed with the pT7-7/Sm-ζ plasmid. Cells were incubated in minimal M9 media at 37°C until they reached an absorbance of 0.7 at 600 nm. Afterward, 1 mM IPTG was added, and cells were incubated at 37°C overnight. The cells were then harvested by centrifugation, and the pellet was stored at −80°C until used. Incubations were performed with continuous shaking at 200 rpm. The M9 minimal media used had [^13^C_6_]-d-glucose (4 g/L) and (0.5 g/L) ^15^NH_4_Cl as sole carbon and nitrogen sources (Sigma-Aldrich), respectively. Purification of the ^13^C, ^15^N uniformly double-labeled Sm−ζ was carried out as described previously for the purification of the non-labeled recombinant ζ subunits.

### Structure determination of Sm-*ζ* by solution NMR spectroscopy

The buffer of the sample was exchanged to 50 mM of NaCl and 25 mM of phosphate buffer at pH 6.8. Next, the sample was concentrated to 1 mM of Sm-*ζ* using a 3 kDa centricon, with 0.5 mM of DSS, 4.5 mM of NaN_3_, and 5% of D_2_O. Afterward, the sample was transferred into the NMR tube and then acquired in a Bruker AVANCE III HD 800 MHz spectrometer for a [^15^N, ^1^H] HSQC (heteronuclear single quantum coherence) spectra (Supplementary Figure S5). The structure of the Sm-*ζ* subunit was determined by solution NMR spectroscopy following the automated J-UNIO protocol (Serrano et al., 2012; Serrano et al., 2014). The backbone NMR experiments 4D APSY-HACANH, 5D APSY-HACACONH, and 5D APSY-CBCACONH were recorded at 293 K on a Bruker AVANCE III HD 800 MHz spectrometer. The sidechain NMR experiments 3D^15^N-resolved, 3D^13^C (aliphatic)-resolved, and 3D^13^C (aromatic)-resolved [^1^H,^1^H]-NOESY experiments were recorded at 293 K with a mixing time of 120 m on a Bruker AVANCE III HD 800 MHz spectrometer. To avoid sample instability, 25% non-uniform sampling was used for each sidechain experiment. The final 20 conformers with the lowest residual CYANA target function values were then subjected to energy minimization. First, each NMR conformer was solvated with full atom TIP3P water containing Cl^−^ and K^+^ ions at ∼0.15 M to mimic the physiological ionic strength. Then, energy minimization and calculation of the conformers were carried out using the GROMACS 2018 (Pronk et al., 2013) package and the Amber14SB (Maier et al., 2015) force field. After the minimization, the energy of the system was computed on each NMR model. In particular, the energy of the protein was computed in a vacuum, while the solvation contribution was computed using Adaptive Poisson–Boltzmann Solver (APBS) software (Jurrus et al., 2018). The final structure is shown in Figures 7, 8, and all structure calculation parameters and statistics are shown in Supplementary Table S2. The structure was deposited in the RCSB PDB with the PDB_id 7VKV and in the Biological Magnetic Resonance Data Bank (BMRB) with the entry 36,447.

### Molecular simulation methods *in silico*

#### Molecular dynamics of the Pd-*ζ* and Sm-*ζ* subunits

The starting structure of Pd-ζ was the PDB_id 2LL0, and that of Sm-ζ was the PDB_id 7VKV. They were processed identically with the *pdb4amber* script, and the starting topology and input coordinate files were created using the LEaP module in AmberTools21 (Case et al., 2005). The AMBER ff99SB (Hornak et al., 2006) force field parameters were used for all the protein residues. Na^+^ counterions were added randomly to neutralize the system, which were then solvated in a truncated octahedron box with explicit TIP3P waters (Jorgensen et al., 1983), with box limits at 10 Å from the protein surface.

The system was minimized with a restraint of 10 kcal mol^−1^ Å^−2^ on all protein atoms, using 1,000 steps of steepest descent followed by 4,000 conjugate gradient steps. Next, the minimized structure was heated from 10 to 298.15 K for 50 ps at constant volume with a 5 kcal mol^−1^ Å^−2^ backbone restraint using the Langevin thermostat with a collision frequency of 5 ps^−1^. Afterward, the system was equilibrated for 500 ps with a 1 atm constant pressure at 298.15 K with a backbone restraint of 1 kcal mol^−1^ Å^−2^, employing the Langevin thermostat and a relaxation time of 5 ps for the Berendsen barostat.

The constant-pH molecular dynamics were performed in triplicate at pH 8.0 for 100 ns with a time step of 2 fs, with all protonable residues allowed to change their protonation states every 200 fs, and 200 fs of solvent relaxation followed any successful protonation changes. Periodic boundary conditions were used, and the particle-mesh Ewald sums (Darden et al., 1993) were employed to treat the electrostatic interactions with a 10 Å cutoff. The SHAKE algorithm (Ryckaert et al., 1977) was used to constrain hydrogen bonds. The GB implicit solvent model (Onufriev et al., 2004) was used during the protonation state change attempts with a salt concentration of 0.1 M. All the simulations were run in GPUs using the *pmemd.cuda* module in Amber20. Each MD simulation was analyzed individually, as shown in Supplementary Material.

## Results

**FIGURE 1:**
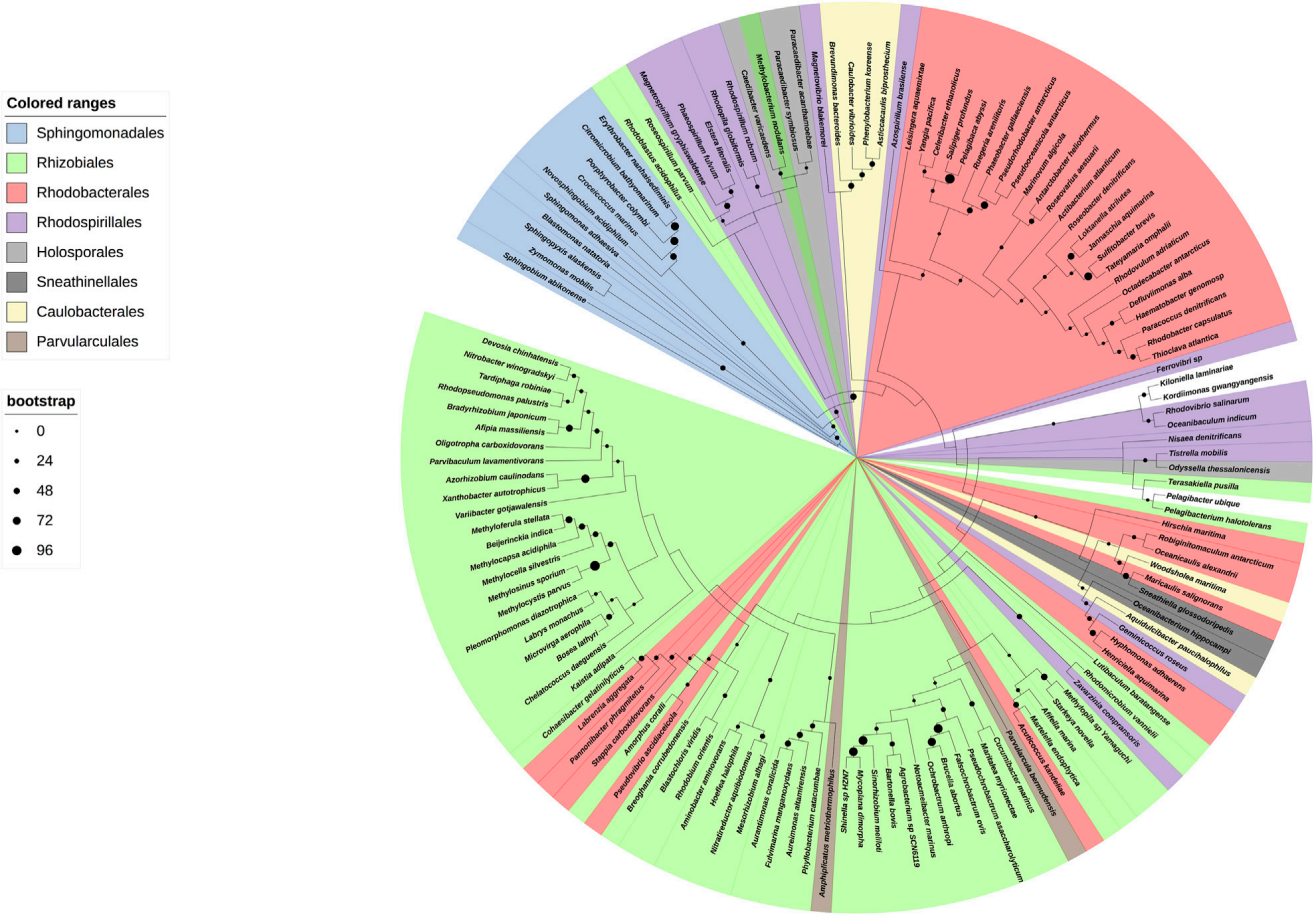
Cladogram of the *ζ* subunit evolution of the *α*-proteobacterial ATP synthases. On the right, a circular cladogram represents the distribution of the *ζ* subunit in the *α*-proteobacteria class. On the left are the orders of the *α*-proteobacteria class that are present in the phylogeny and their color coding. On the bottom left, the bootstrap values are shown by circles of different sizes. The cladogram shows how the *ζ* subunit distributes in the different orders of the α-proteobacteria; in a few cases, such as some Rhodobacterales and Rhizobiales, the *ζ* sequences are grouped outside of their orders, likely as a result of small gene size or horizontal transfer. The Rhodospirillales are grouped into two different branches of the phylogeny. The *ζ* sequences of a representative from the orders Pelagibacterales, Klioniellales, and Kordiimonadales, which are *Pelagibacter ubique*, *Klionella laminariae*, and *Kordiimonas gwangyangensis*, respectively, are not color-coded.

We started with a preparative bioinformatic analysis of the ζ and ε subunits of the ATP synthases of α-proteobacteria to update sequence files and alignments and to confirm the fact that the main inhibitory function of the F_1_F_O_-ATPase in α-proteobacteria was lost in ε and it was acquired by ζ in α-proteobacteria (see the first section of Supplementary Material and Supplementary Figures S1, S1.1). Afterward, with the role of ζ as the main inhibitor of the α-proteobacterial F-ATPase already established, we focused on the evolution of ζ along several α-proteobacterial species to define whether the inhibitory function of ζ is preserved or not all along the α-proteobacteria class. This also aimed to study the evolution of this subunit in relation to the close relationship of α-proteobacteria with the endosymbiotic origin of mitochondria (Archibald, 2015; Ku et al., 2015). We carried out a comprehensive phylogenetic analysis of ζ, as described in the Supplementary Material, all along the α-proteobacteria class. In the resulting phylogeny, closest to the ζ gene of *P. denitrificans* (αPATPsζ) (Mendoza-Hoffmann et al., 2022) (around 2:30 o’clock of the circular ζ′s phylogeny, Figure 1), there are some members of the *Paracocccus* genus and some photosynthetic α-proteobacteria, such as *Rhodobacter capsulatus* and *Rhodobacter sphaeroides* (now renamed *Cereibacter sphaeroides* (Hördt et al., 2020)), and in some of them, we confirmed the conservation of the inhibitory function of ζ (see the following section). In close proximity to *P. denitrificans* in the Rhodobaterales order, we also find some marine α-proteobacteria, such as *Jannaschia sp.*, in which we previously confirmed its heterologous inhibitory function (Zarco-Zavala et al., 2014). Around 6:00 o’clock of the ζ cladogram (Figure 1), there are some nitrogen-fixing Rhizobiales, which are more distant to our reference *P. denitrificans* ζ (Pd-ζ), which were also studied here (see the following section). In this ζ cladogram, some ζ sequences branched outside their respective orders, as in some Rhodobacterales and Rhizobiales, but these inconsistencies were corrected when rRNAs were used for the cladogram construction (see Figure 9), so these discrepancies are likely a result of horizontal transfer or small gene size.

### Conservation and evolution of the ζ subunit in photosynthetic and marine α-proteobacteria compared with the ζ subunit of *P. denitrificans*

**FIGURE 2:**
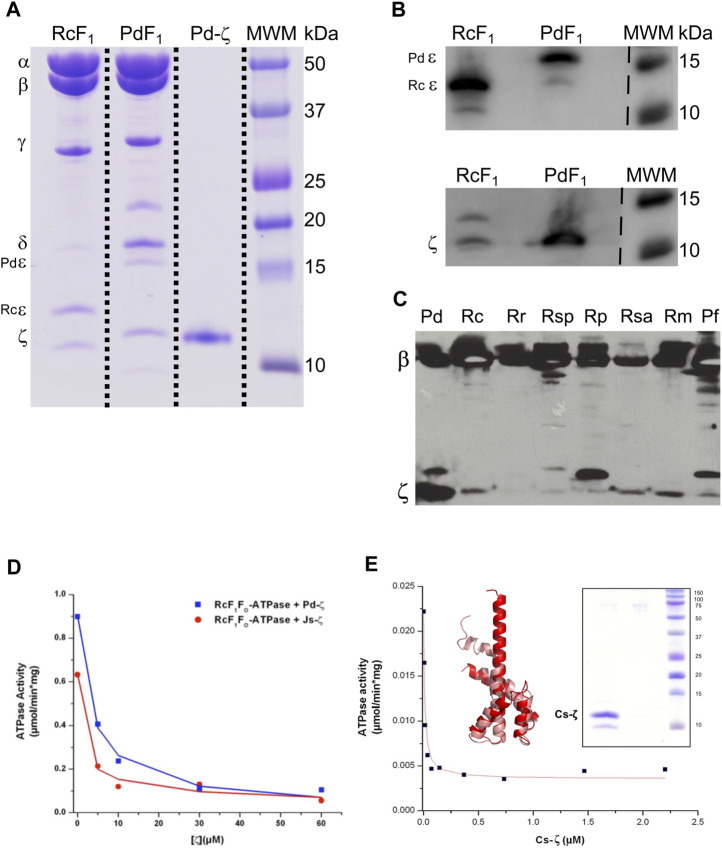
Purification and inhibition of the F_1_-ATPase from *R. capsulatus* and the F_1_F_O_-ATPase of *C*. *sphaeroides*. **(A)** Coomassie-stained SDS-PAGE of the purification of *R. capsulatus* F_1_ (F_1_Rc). All the subunits of the Rc F_1_ are present: *α*, *β*, *γ*, *δ*, *ε*, and *ζ*. The subunit Rc-ε has a molecular weight of 13 kDa, which is lower than that of Pd-*ε* (15.8 kDa). **(B)** Detection of subunits Rc-ε and Rc-*ζ* by WB. The upper and lower images are the same PVDF membrane; it was revealed first as the bottom one (anti-ζ), and after stripping, it was exposed to anti-ε antibodies and revealed again. These two images were spliced where indicated by discontinuous lines to remove empty lanes. **(C)** WB anti-*β* and anti-*ζ* of the enriched F_1_-ATPases by the chromatophore’s chloroform extraction from photosynthetic α-proteobacteria; the whole PVDF membrane was incubated with both antibodies; this image was not spliced. **(D)** The F_1_F_O_ complex of *R. capsulatus* (RcF_1_F_O_) was solubilized from chromatophores. This F_1_F_O_ complex was preincubated in the presence of increasing concentrations of the recombinant Pd-ζ (blue) or the recombinant ζ of *Jannaschia* sp*.* (Js-ζ, red). Afterward, the RcF_1_F_O_-ATPase activity was determined by the coupled ATPase assay as described in Materials and methods. Lines represent the non-linear fitting to a non-competitive inhibitor model, and average data of duplicate experiments are shown. **(E)** The recombinant Cs-ζ was reconstituted into chromatophores of *C. sphaeroides*, and the inhibition of the CsF_1_F_O_-ATPase by Cs-ζ was determined by the same coupled ATPase assay. The plot shows a representative experiment of three independent ones. Curve fitting was made as before and the _app_IC_50_ of Cs-ζ to inhibit its own CsF_1_F_O_-ATPase was 9.7 ± 2.7 nM (average ±SD). Inset left: the Cs-ζ structure modeled by Phyre2 and refined in Swiss-Model (salmon) or by AlphaFold (red) with respective identities of 100% and 90% to our cloned Cs-ζ protein. Inset right: Coomassie-stained SDS-PAGE gel of the purified Cs-ζ.

We started with functional studies of the ζ subunits by cloning and overexpressing the recombinant ζs from some of the aforementioned α-proteobacteria, initially with the purification of the F_1_-ATPase from photosynthetic α-proteobacteria, closely related to *P. denitrificans* in the same Rhodobacterales order. The ATP synthases from some of these photosynthetic α-proteobacteria were functionally characterized (Turina et al., 1992; Maldonado et al., 1998; Feniouk et al., 1999; Feniouk et al., 2002; Turina et al., 2004; Feniouk et al., 2005). Although we previously observed the ζ subunit of *C. sphaeroides* (Cs-ζ) bound to its CsF_1_F_O_-ATP synthase of chromatophores (Morales-Rios et al., 2010), we did not confirm that Cs-ζ inhibited its own ATP synthase. The close relationship between *P. denitrificans* and *C. sphaeroides* (Figures 1, 9) strongly suggests that the inhibitory function of ζ should be conserved in Cs-ζ. Together with *C. sphaeroides*, another closely related α-proteobacteria to *P. denitrificans* is *Rhodobacter capsulatus* (Zarco-Zavala et al., 2014) (Figures 1 and Supplementary Figure S1), suggesting that the ATP synthase of *R. capsulatus* (RcF_1_F_O_-ATPase) should harbor the ζ-binding site at the INGECORE or α_DP_β_DP_γ interface (Mendoza-Hoffmann et al., 2018a, 2018b; Zarco-Zavala et al., 2018) of the Rc-F_1_F_O_ ATPase. Therefore, we purified the F_1_-ATPase of *R. capsulatus* (RcF_1_), obtaining a functional F_1_-ATPase with the canonical α, β, γ, δ, and ε subunits and a sixth 11 kDa subunit migrating similar to Pd-ζ of Pd-F_1_ (Morales-Rios et al., 2010), below a 15 kDa subunit presumably being Rc-ε (Figure 2A). The identity of these subunits as Rc-ε and Rc-ζ were confirmed by Western blot analyses carried out separately with monoclonal anti-ε (Figure 2B upper panel) and polyclonal anti-ζ (Figure 2B lower panel) antibodies.

To confirm the presence of ζ in other photosynthetic α-proteobacteria, we analyzed the chromatophores of several of these species, including *Rhodospirillum marinarum* (Rm), *Rhodopseudomonas palustris* (Rp), *Rhodovibrio salinarum* (Rsa), *Rhodospirillum rubrum* (Rr)*, Rhodobacter capsulatus* (Rc), and *Phaeospirillum fulvum* (Pf). The anti-β and anti-ζ Western blot analyses confirmed the presence of ζ in chromatophores of all of these photosynthetic α-proteobacteria, besides the presence of the catalytic *ß* subunit (Figure 2C). Some of these ζ bands were relatively weak compared to that of *P. denitrificans* (first lane of Figure 2C). The weakness of these signals could be the result of a lower affinity of the antibody anti-ζ for the ζ subunits from relatively distant photosynthetic α-proteobacteria. For instance, the ζ subunit of *R. rubrum* (Rr-ζ) is one of the most distant to Pdζ (Supplementary Figure S1A, and Figure 9 around 2 o’clock). In summary, these results show that ζ is expressed and spread as α-proteobacterial F-ATPase inhibitor along most, if not all, photosynthetic α-proteobacteria.

To confirm the inhibitory function of ζ in photosynthetic and non-photosynthetic α-proteobacteria, we performed heterologous reconstitution of the available recombinant ζ subunits from *P. denitrificans* (Pd-ζ) and *Jannaschia sp.* (Js-ζ) into the ATP synthase from photosynthetic α-proteobacteria. The latter is another marine α-proteobacteria of the Rhodobacterales order, also closely related to *P. denitrificans* (see Figures 1, 10 and Supplementary Figure S1A). It is worth recalling that in previous experiments, Js-ζ was able to inhibit the PdF_1_-ATPase (Zarco-Zavala et al., 2014). With these antecedents, we carried out heterologous reconstitution of Pd-ζ and Js-ζ into the F_1_F_O_-ATPase of *R. capsulatus* (Rc-F_1_F_O_) solubilized from chromatophores. As expected, from their close proximity (Zarco-Zavala et al. (2014) and Figure 1 and Supplementary Figure S1A), we observed a potent inhibitory activity of Pd-ζ and Js-ζ subunits on the RcF_1_F_O_-ATPase (Figure 2D). These results showed that both Pd-ζ and Js-ζ subunits can bind productively to the INGECORE binding site of Rc-F_1_-ATPase. The _app_IC_50_ of the Pd-ζ to inhibit the RcF_1_F_O_-ATPase obtained was 3.76 μM, and the corresponding _app_IC_50_ of Js-ζ was 1.12 μM. These _app_IC_50_ values are, as expected from heterologous reconstitutions, higher than the _app_IC_50_ of Pd-ζ of 0.44–0.55 μM (see Supplementary Material and the work of Morales-Rios (2011)) to inhibit its own PdF_1_F_O_-ATPase in SBP*.*

In parallel, we were able to clone, overexpress, and purify the recombinant ζ subunit from *C. sphaeroides* (Cs-ζ) (Figure 2E, inset) and reconstituted it into the CsF_1_F_O_-ATP synthase of *C. sphaeroides* chromatophores. Interestingly, we obtained an average _app_IC_50_ of 9.7 ± 2.7 nM of the Cs-ζ to inhibit its own CsF_1_F_O_-ATPase in chromatophores after three independent determinations (Figure 2E shows a representative curve). This value is in the nM range, much lower than the observed _app_IC_50_ of Pd-ζ (or Js-ζ) to inhibit the PdF_1_F_O_-ATPase in PSB, which are in the μM range. Recently, three new AlphaFold (AF)-modeled structures of the Cs-ζ protein were uploaded, and the structure closest to our cloned Cs-ζ protein was 90% identical (AF-A0A3G6WKK1-F1-model-v4. pdb). We aligned this AF-Cs-ζ model (red in Figure 2E, inset) with our own model of the cloned Cs-ζ constructed in Phyre2 and refined in SWISS-MODEL (salmon in Figure 2E, inset). Our Cs-ζ model shows, as expected, the Pd-ζ-like structure with the inhibitory N-terminus was partially disordered; however, the AF-Cs-ζ model shows the N-terminus with the inhibitory extended α-helix conformation (Figure 2E, inset). As in other ζ structures described in the following text, this shows that the transition from the disordered to the ordered α-helical extended inhibitory structure of Pd-ζ may take place in Cs-ζ, as confirmed here experimentally (Figure 2E). In summary, the Cs-ζ is the ζ subunit of α-proteobacteria showing the highest affinity for its target homologous F_1_F_O_-ATPase found so far (Figure 2E), even higher than the affinity of Pd-ζ or Js-ζ (Zarco-Zavala et al., 2014). As we will see, these high-affinity ζ subunits are found in free-living α-proteobacteria subjected to extreme environmental changes. Thus, they require a high-affinity and strongly inhibitory ζ subunit of the F_1_F_O_-ATPase to prevent futile ATP consumption and cope with the variable, thus challenging living environments that face α-proteobacteria.

Unfortunately, we could not grow *Jannaschia sp*. (kindly donated by Prof. Mary Ann Moran) to estimate the _app_IC_50_ of Js-ζ for its own Js-ATP synthase. Nevertheless, it is evident that in free-living α-proteobacteria, the ζ subunit is a potent F_1_F_O_-ATPase inhibitor with nM or μM affinities for their respective or homologous free-living α-proteobacterial F_1_F_O_-ATPases. Although we were unable to clone the recombinant ζ from *R. capsulatus* (Rc-ζ) and to estimate its _app_IC_50_ to inhibit its own Rc-ATP synthase, we consider that Rc-ζ is likely a potent RcF_1_F_O_-ATPase inhibitor. This is because of the very close relationship of *R. capsulatus* with *P. denitrificans*, *C. sphaeroides*, and *Jannaschia sp.* (see Figures 1, 10; Supplementary Figure S1) and the previous results showing the strong conservation of the ζ inhibitory function in free-living α-proteobacteria. It was also important to assess this functional conservation of ζ in more distant and free-living photosynthetic α-proteobacteria, such as *Rhodospirillum rubrum* (Rr-ζ). This α-proteobacterium belongs to the order Rhodospirillales but not to the Rhodobacterales (Figures 1, 9). The sequence of Rr-ζ is more divergent from the Pd-ζ than the previous ζ′s of Rhodobacterales (see Supplementary Figure S1A).

**FIGURE 3:**
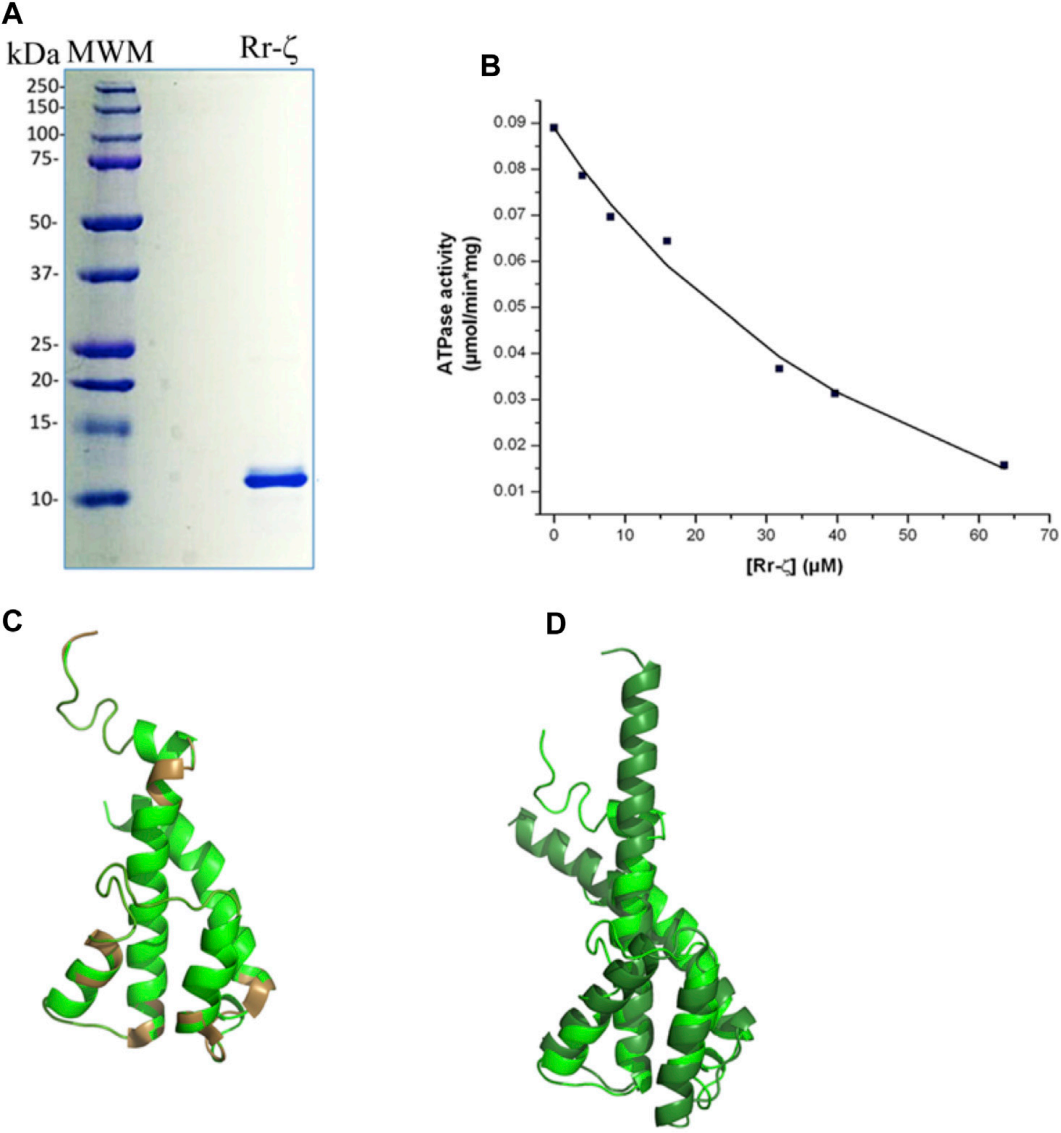
Purification, reconstitution, and structure of the Rr-ζ subunit. **(A)** SDS-PAGE of the purified Rr-ζ subunit. **(B)** Reconstitution and effect of the Rr-ζ subunit on the RcF_1_F_O_-ATPase activity of chromatophores. The shown amounts of pure Rr-ζ were reconstituted into the *R. rubrum* chromatophores, and subsequently, the RcF_1_F_O_-ATPase was determined as described in Materials and methods. The plot shows a non-linear curve fit to a non-competitive inhibition model of a representative experiment of at least three replications (see text for further statistical details). **(C)** Structure of a homology model of the Rr-ζ subunit (green) constructed in Phyre2 which showed the lowest RMSD compared with the most representative NMR structure of Pd-ζ (PDB_id 2LL0) superimposed in brown. This Rr-ζ model shows the intrinsically disordered protein region (IDPr) of the inhibitory Rr-ζ N-terminus. **(D)** Superposition of the AF model of the ζ subunit of *Rhodospirillales bacterium* (darker green) with the Rr-ζ model of **(C)**. The AF model shows the inhibitory extended N-terminal α-helical conformation of ζ that blocks the F_1_-ATPase rotation. See text for further details.

To assess the homologous inhibitory capacity of Rr-ζ on chromatophores from *R. rubrum*, we overexpressed and purified the Rr-ζ subunit (Figure 3A) and reconstituted it into the RrF_1_F_O_-ATPase in conditions promoting the productive binding of the Rr-ζ by the entrance rotation alpha helix locking (ERAHL) mechanism (Mendoza-Hoffmann et al., 2018a, 2018b; Zarco-Zavala et al., 2018). These ATPase assays showed a clear dose-dependent Rr-ζ inhibition of the Rr-F_1_F_O_-ATPase of chromatophores with an _app_IC_50_ of 18.4 ± 8.2 μM on average, after three independent determinations, and fitting the data to a non-competitive inhibition mechanism (see the representative curve in Figure 3B) (Zarco-Zavala et al., 2014). This _app_IC_50_ of Rr-ζ to inhibit its own Rr-ATP synthase is about 35-fold higher than the _app_IC_50_ of the Pd-ζ (0.55 μM) to inhibit its own PdF_1_F_O_-ATPase complex in SBP (see Figure 6D) and ≈1,900-fold higher than that of Cs-ζ (9.7 nM, see the previous reference). This shows clearly that the inhibitory function of ζ is still preserved not only in the Rhodobacterales order (Figure 1 and Supplementary Figure S1) but also in the Rhodospirillales order. However, it is worth emphasizing that this Rr-ζ inhibitory function has a very low affinity compared to Pd-ζ or Cs-ζ. In addition, we also tried to obtain a heterologous inhibition of the Rr-ζ into the PdF_1_F_O_-ATPase and PdF_1_-ATPase complexes of *P. denitrificans*. However, the results never showed any inhibitory activity of Rr-ζ on PdF_1_F_O_-ATPase using a large excess of 30 μg of Rr-ζ in SBP from *P. denitrificans* (data not shown).

These observations indicate that the ζ subunit of photosynthetic free-living α-proteobacteria, such as *C. sphaeroides*, and the more distant *R. rubrum* ζ subunits preserve their inhibitory function, although the latter exhibits a relatively much lower affinity. The data, thus, show a tendency to lose the inhibitory function of ζ in α-proteobacteria that are more distant from *P. denitrificans*.

We also tried to correlate this preserved inhibitory function of the “photosynthetic” ζ subunits with their structure. In the first approach, we modeled the structure of Rr-ζ by homology with the Pd-ζ subunit preserving as expected, essentially the same ζ fold as Pd-ζ, with the inhibitory N-terminus intrinsically disordered, and the globular domain folded as four-α-helix bundle (Figure 3C). The Rr-ζ is slightly larger than the Pd-ζ (Supplementary Figure S1A) due to a 3-aa insertion in the loop between α-helices 3 and 4 (at position 81 in the numbering o Pd-ζ); this is depicted as a brown loop (bottom of Figure 3C) that does not align well with Pd-ζ (Rr-ζ in green in Figure 3C). There is also a single 1-aa insertion in position four of the N-terminus of Rr-ζ compared with Pd-ζ, and it is also clear that the N-terminus of Rr-ζ is more divergent from Pd-ζ than the other Rhodobacterales ζ-subunits closer to *P. denitrificans* (Supplementary Figure S1A). These results explain why Rr-ζ is still able to inhibit, although weakly, its own RrF_1_F_O_-ATPase but unable to inhibit the PdF_1_F_O_-ATPase heterologously, most likely because of the divergent N-terminus of Rr-ζ (Supplementary Figure S1A).

In a second approach to look for a structure–function correlation of the Rr-ζ, we further investigated the ζ structures modeled in AF named DUF 1476, finding 22 structures of ζs from Rhodospirillaceae *bacterium* within the Rhodospirillales order and very closely related to Rr-ζ (see the following link: https://alphafold.ebi.ac.uk/search/text/duf1476? organismScientific Name=Rhodospirillaceae bacterium). Within these 22 structures, we found one with a higher identity to Rr-ζ named AF-A0A7V7E8W8-F_1_-model_v4. pdb. This structure, among others, was aligned to our model of Rr-ζ, and we found that the RMSD between this AF model and our Rr-ζ model was the smaller one (2.034 Å), as calculated in PyMol (Figure 3D). Other AF Rhodospirillaceae *bacterium* models had higher RMSD and lower identity than our Rr-ζ model. In the structural alignment (Figure 3D), the AF-A0A7V7E8W8-F_1_-model_v4. pdb was longer than Rr-ζ in the C-terminus. Still, its inhibitory N-terminus is similar to the Rr-ζ. The full identity between our model and the AF model is 38%, confirming them as closely related orthologous proteins. Interestingly, as found with other AF ζ structures, the AF Rhodospirillaceae-ζ structure does not show the intrinsically disordered N-terminus as in the Pd-ζ NMR structure (PDB_id 2LL0), but the inhibitory extended α-helical N-terminus that blocks the rotation of the central rotor of the PdF_1_ and PdF_1_F_O_-ATPases (Morales-Rios et al., 2015; Garcia-Trejo et al., 2016; Mendoza-Hoffmann et al., 2018a, 2018b; Zarco-Zavala et al., 2018). Although AF does not show the experimental (NMR, X-ray, or Cryo-EM) protein structures, it shows some putative conformers of the protein of interest as predicted from the available experimental structures (Jumper et al., 2021). Therefore, it seems worth considering that the AF extended α-helical N-terminal structures represent the inhibitory ζ conformations that might be reached in the appropriate conditions, either spontaneously in solution or after its productive binding to a compatible F_1_-ATPase. With these considerations, the AF-modeled ζ structures closer to the Rr-ζ structure show that these ζ subunits may, therefore, reach the extended α-helical N-terminus inhibitory conformation. This interpretation is in full concordance with the inhibitory function of Rr-ζ working on its own RrF_1_F_O_-ATPase (Figure 3B). In summary, these data show that the ζ subunits of marine and photosynthetic α-proteobacteria are conserved enough to be able to undergo the inhibitory N-terminal transition from non-inhibitory intrinsically disordered (IDPr) to the extended α-helical structure to inhibit unidirectionally the α-proteobacterial F_1_F_O_-ATPase.

### Evolution of the ζ subunit in facultative symbiotic, facultative pathogenic, and strictly parasitic α-proteobacteria

**FIGURE 4:**
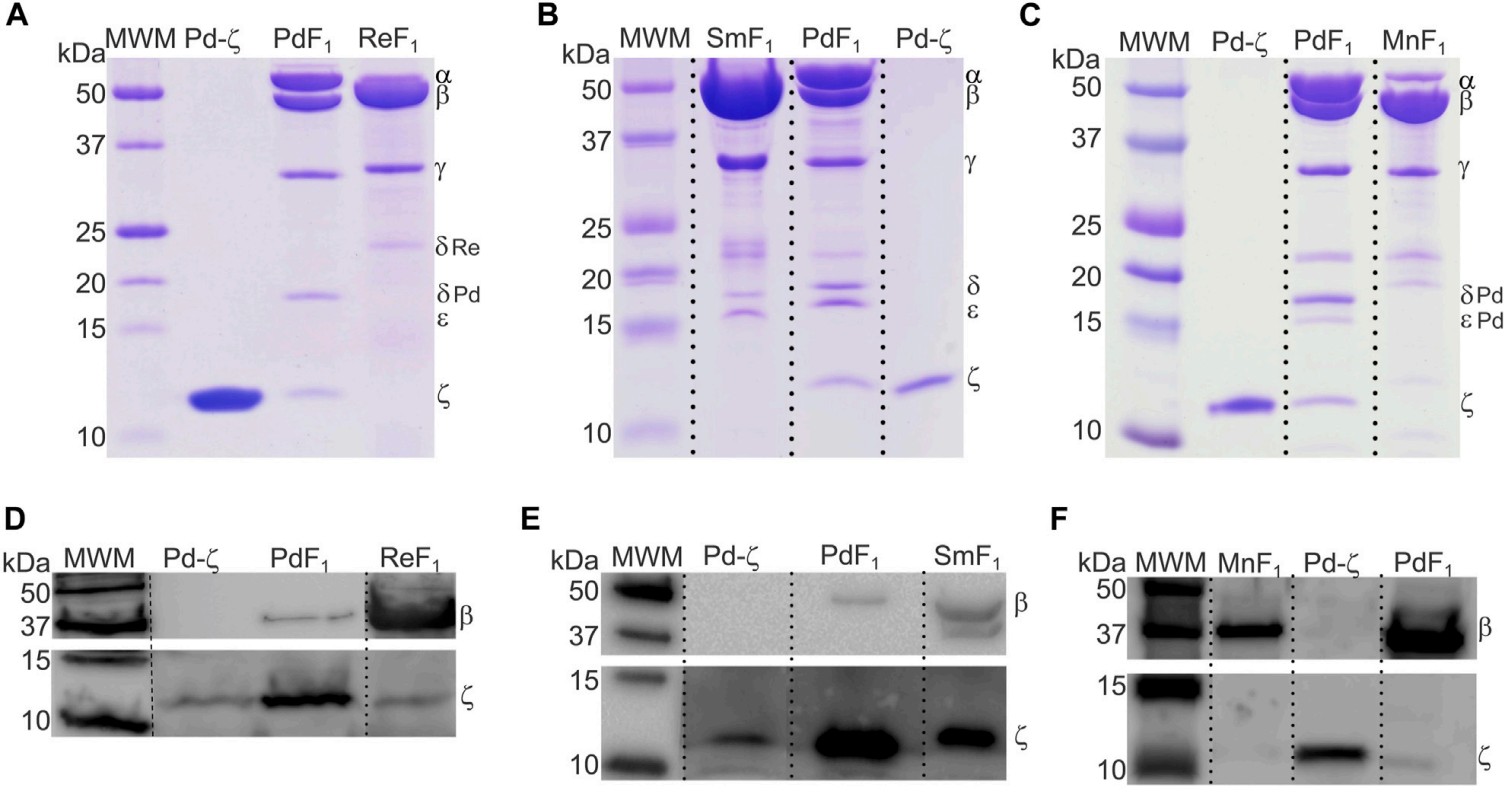
The ζ-gene is expressed and associated with the F_1_ complex of *R. etli*, *S. meliloti*, and *M. nodulans*. **(A–C)** Coomassie-stained SDS-PAGE of the purifications of F_1_-ATPases from *R. etli* (Re), *S. meliloti* (Sm), and *M. nodulans* (Mn), respectively. **(D–F)** WB anti-β (upper images) and anti-ζ (lower images) of the F_1_ complexes of Re, Sm, and Mn, respectively. The *ζ* subunit is detected as faint bands in all Rhizobiales F_1_-ATPases, with the exception of *S. meliloti,* where Sm-ζ is a more prominent band. All images were spliced to remove empty lanes, indicated by vertical discontinuous lines.

So far, we described the functional and structural properties of the ζ subunits from free-living α-proteobacteria closely (Rhodobacterales) and distantly (Rodospirillales) related to Pd-ζ, confirming, perhaps non-unexpectedly, the conservation of the inhibitory function of these ζ subunits. We, thus, turned our focus to the ζ subunits of symbiotic, pathogenic, and parasitic α-proteobacteria in order to define whether the inhibitory function of ζ is preserved in α-proteobacteria of different lifestyles, either closely or distantly related to *P. denitrificans*. To this aim, we isolated and functionally characterized the ζ subunits and F_1_- or F_1_F_O_-ATPases from facultative nitrogen-fixing symbiotic Rhizobiales α-proteobacteria (green in Figures 1, 9). The F_1_-ATPases were isolated first from *R. etli*, *S. meliloti*, and *Methylobacterium nodulans*. All of these are facultative symbionts of legume plant roots, where they exchange nutrients for fixed nitrogen with legume plants. We grew these bacteria aerobically in rich LB media and isolated the F_1_-ATPase from inverted membranes, prepared as described in *Materials and methods*. The isolation of the F_1_-ATPases from these Rhizobiales α-proteobacteria produced the canonical F_1_-ATPase pattern of the five protein bands in Coomassie-stained SDS-PAGE gels, namely, the *α*, *β*, *γ*, *δ*, and ε subunits, but we could not see clearly the sixth ζ band of the PdF_1_-ATPase (see Morales-Rios et al. (2010) and Zarco-Zavala et al. (2014) and the third lanes of Figures 2A, 4A–C). We considered that ζ was either absent or present in very low amounts, non-detectable by Coomassie staining. Therefore, we carried out immunodetection by anti-ζ Western blot analyses, with our polyclonal anti-Pd-ζ antibody (Morales-Rios et al., 2010), including a control with a monoclonal anti-β. The results showed clearly the presence of trace amounts of ζ in all F_1_-ATPases of Rhizobiales α-proteobacteria, i.e., *R. etli* (F_1_Re), *S. meliloti* (F_1_Sm), and *M. nodulans* (F_1_Mn), in addition to the presence of the control *ß* subunit (Figures 4D–F). However, the ζ band intensities were weak compared with the Pd-ζ band (F_1_ Pd). Although this might be due to the sequence diversity of the Rhizobiales ζ subunits, the lack of a clear ζ band intensity in the Coomassie-stained gels (Figures 4A–C) evidences that ζ is present in very low amounts in Rhizobiales F_1_-ATPases. This indicates that either the ζ subunits of Rhizobiales are sub-expressed at low levels and/or they have a lower affinity for their respective F_1_-ATPases, thus becoming dissociated from the soluble F_1_-ATPase during the purification of the enzyme.

**FIGURE 5:**
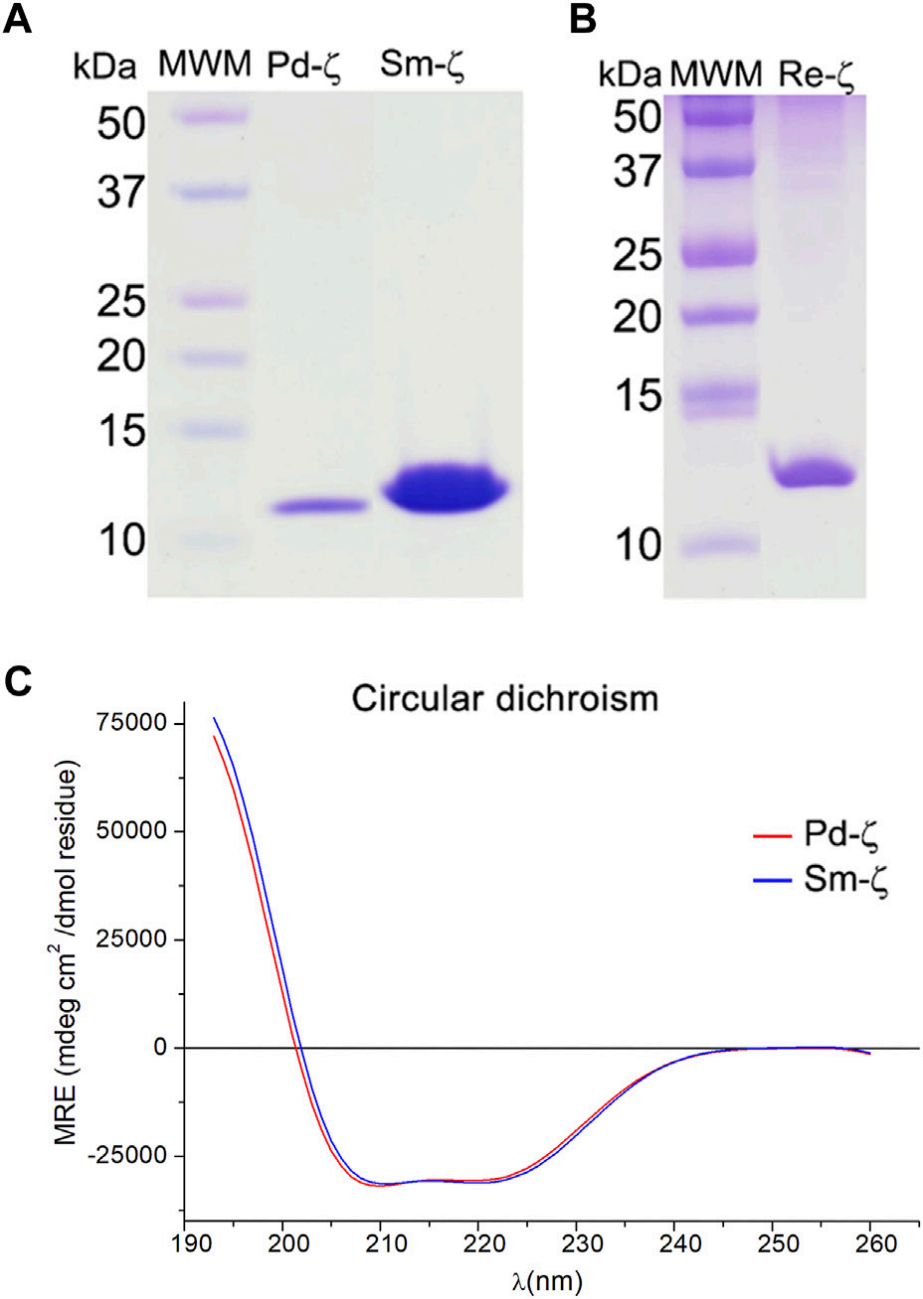
Purified recombinant ζ subunits from *R. etli* and *S. meliloti*. **(A, B)** Coomassie blue-stained SDS-PAGE **(A)** from left to right: the molecular weight marker (MWM), recombinants Pd-ζ and Sm-ζ; **(B)** from left to right: molecular weight marker (MWM), recombinant Re-ζ. **(C)** Far UV CD spectra of 0.07 mg/ml of Pd-ζ (red) and Sm-ζ (blue) diluted in 25 mM of phosphate buffer with pH 8.0. See Materials and methods for details.

To assess these possibilities, we cloned the recombinant ζ subunits from *S. meliloti* (Sm-ζ) and *R. etli* (Re-ζ) chromosomic DNA to determine the _app_IC_50_ of these Rhizobiales ζ subunits to inhibit their F_1_ and/or F_1_F_O_-ATPase complexes. The recombinant Sm-ζ and Re-ζ subunits were purified as described previously for Pd-ζ (Morales-Rios et al., 2010; Zarco-Zavala et al., 2014) to achieve high purity (>95% according to Coomassie staining, Figures 5A,B). The highest yield and purity were always obtained with the Sm-ζ (Figures 5A, B). In addition, the Sm-ζ was also the more intense band in anti-ζ Western blots compared with the homologous Re-ζ and Mn-ζ subunits (Figures 4D–F); therefore, further functional and structural studies of Rhizobiales ζs were carried out with Sm-ζ.

Before carrying out the functional analyses with Sm-ζ, we confirmed the proper folding of the protein by the content of α-helical secondary structure determined by circular dichroism (CD) spectroscopy, compared with Pd-ζ as the reference. As can be seen in Figure 5C, the CD spectra of Pd-ζ and Sm-ζ subunits are almost identical and superimpose very well to each other, indicating that in solution, the α-helical content of the Sm-ζ is very similar to that of Pd-ζ. This indicates the proper folding of the Sm-ζ, at least as the overall α-helical content, suggesting that the globular part of Sm-ζ is folded similarly as the native Pd-ζ subunit in solution (see PDB_id 2LL0 and Figure 8A). We calculated the α-helical content from the CD data of the Pd-ζ and Sm-ζ subunits as described in *Materials and methods*. In summary, the estimated α-helical contents of Pd-ζ and Sm-ζ were 74.8% and 75.7%, respectively, i.e., very close values indicating a similar amount of α-helix in both proteins, as in the Pd-ζ subunit (see PDB_id 2LL0, the work of Zarco-Zavala et al. (2014), and Figure 8A).

**FIGURE 6:**
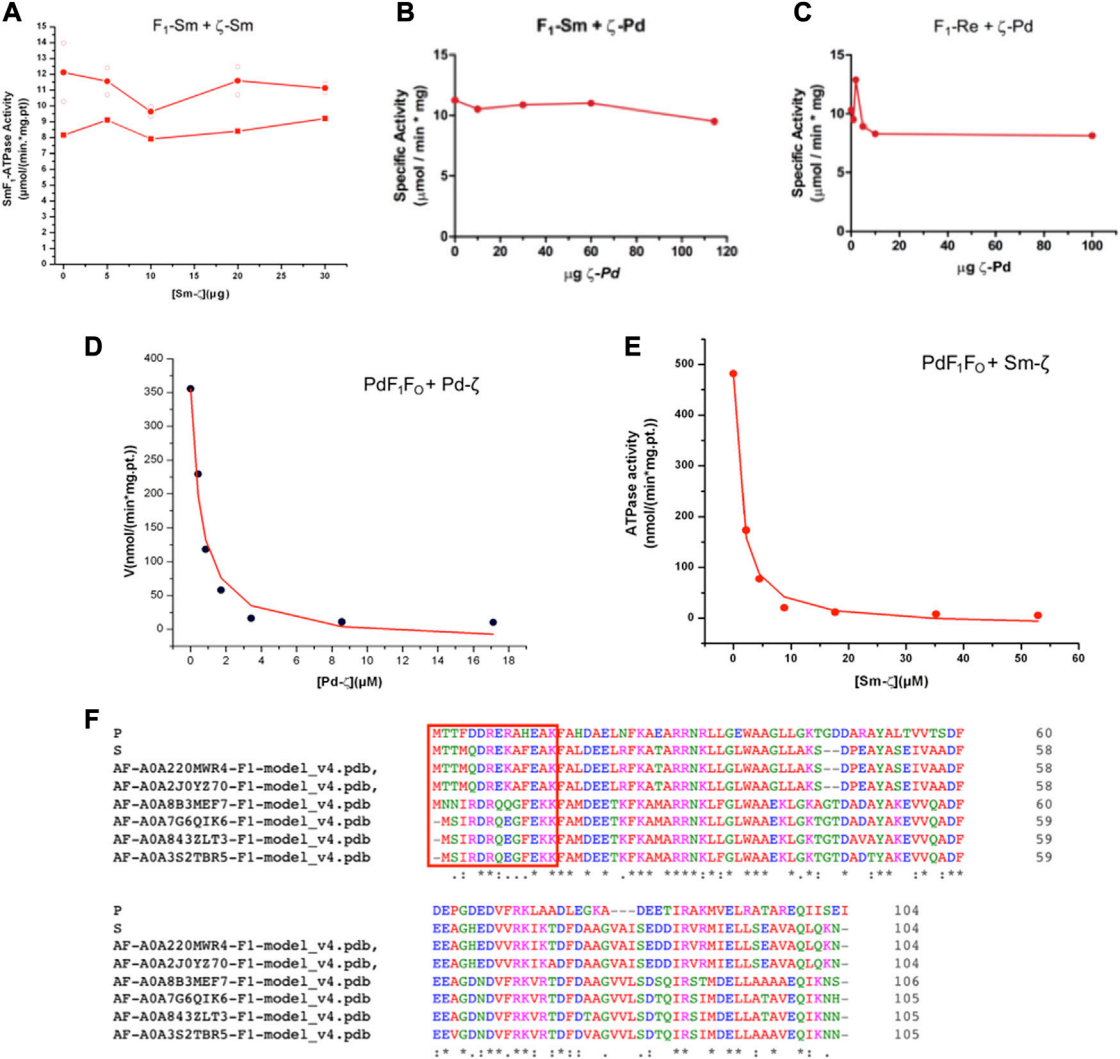
Effect of Sm-ζ and Pd-ζ on the F_1_-ATPase or F_1_F_O_-ATPase activities after homologous or heterologous reconstitutions. **(A)** Homologous reconstitution of *Sinorhizobium meliloti* F_1_ (SmF_1_) with increasing concentrations of its own recombinant Sm-ζ. Individual identical duplicate experiments with 20 min of preincubation of Sm-ζ with SmF_1_ as described in Material and methods are shown in open circles (**O**), with their average in red-filled circles (●). A third non-identical experiment with 24 h preincubation with Sm-ζ and SmF_1_ is shown in red-filled squares (■). There was no inhibition at all of SMF_1_ by Sm-ζ in any case. **(B)** Heterologous reconstitution of SmF_1_ with increasing amounts of recombinant Pd-ζ. **(C)** Heterologous reconstitution of *Rhizobium etli* F_1_ (ReF_1_) with increasing quantities of recombinant Pd-ζ. **(B, C)** We added a major excess of Pd-ζ or Sm-ζ (≥100 μg) to confirm the non-inhibitory effects instead of repeating these experiments. **(D)** Homologous reconstitution of increasing amounts of recombinant Pd-ζ on the PdF_1_F_O_ of SBP^*Δζ*^, showing a representative experiment of three independent ones; the curve is a fit to the non-competitive inhibitor model (●), and the _app_IC_50_ obtained for Pd-ζ is on average 0.55 ± 0.38 μM (±SD). **(E)** Heterologous reconstitution of the Sm-ζ on the PdF_1_F_O_-ATPase of SBP^*Δζ*^; representative experiment of three independent ones, a similar fitting as in **(D)** gave an average _app_IC_50_ of 1.45 ± 0.38 μM (±SD) (●). The _app_IC_50_ of Sm-ζ is significantly higher by 2.6-fold than that of Pd-ζ (see text and Supplementary Figure S2B).

Once confirmed that Sm-ζ is folded similarly to the Pd-ζ subunit, we carried out the homologous reconstitution of Sm-ζ into the isolated SmF_1_-ATPase, which also showed more clearly the contents of α, β, γ, δ, and ε subunits better than other Rhizobial F_1_-ATPases (see Figures 4A–C). This enzyme showed an SmF_1_-ATPase activity (11.24 ± 0.79 μmol/(min.*mg.pt.)) higher than that of PdF_1_^Δζ^ (4.48 ± 1.21 μmol/min*mg pt), which is with the PdF_1_^Δζ^ isolated from the ζ knockout mutant PdΔζ (Mendoza-Hoffmann et al., 2018) and MnF_1_ (5.53 ± 0.03 μmol/(min*mg.pt.)) ATPases and similar to that of ReF_1_ (10.37 ± 2.57 μmol/(min.*mg.pt.)). Therefore, our SmF_1_ was an optimal preparation to assess the inhibitory function of Sm-ζ since it lacked most of its endogenous Sm-ζ and, thus, showed one of the highest F_1_-ATPase activities of the α-proteobacterial F_1_-ATPases isolated here. Somehow unexpectedly, we observed no inhibitory effect of the Sm-ζ whatsoever on its own SmF_1_-ATPase after reconstitution experiments with increasing concentrations to reach an excess of Sm-ζ up to 30 μg in the presence of MgATP (see Figure 6A) in conditions where the Pd-ζ inhibits fully the PdF_1_ATPase and PdF_1_F_O_-ATPase activities (Mendoza-Hoffmann et al., 2018; Zarco-Zavala et al., 2018). After repeating this in an identical duplicated experiment (see red circles in Figure 6A), observing no inhibitory function of Sm-ζ on the SmF_1_-ATPase on average, we carried out a non-identical replication of this experiment extending the preincubation of Sm-ζ with SmF_1_ by 24 h instead of 20 min (as described in *Materials and methods*) to make sure there was enough time to reveal any inhibitory function of Sm-ζ on SmF_1_, in case there were any (red squares in Figure 6A). Once more, there was no inhibitory function of Sm-ζ on the SmF_1_-ATPase whatsoever. Given this non-inhibitory result, we assayed the putative heterologous inhibitory function of Sm-ζ on the PdF_1_-ATPase. Surprisingly, we observed a strong inhibition of the PdF_1_-ATPase after reconstitution with large amounts (15–20 μg) of Sm-ζ (Supplementary Figure S2A). To confirm these results, we used the full PdF_1_F_O_^Δζ^-ATPase of SBP lacking the endogenous ζ (Mendoza-Hoffmann et al., 2018a, 2018b) to estimate an _app_IC_50_. With this experiment (Figure 6E), we confirmed that the Sm-ζ exerted a dose-dependent strong inhibition of the PdF_1_F_O_^Δζ^-ATPase of SBP. We estimated an _app_IC_50_ after repeating this experiment three times and obtained an average _app_IC_50_ value of 1.45 ± 0.38 μM after adjusting the data points to the non-competitive inhibition model (see Figure 6E). This value is 2.6-fold higher than the _app_IC_50_ of Pd-ζ (0.55 ± 0.36 μM) estimated in the PdF_1_F_O_-ATPase of SBP with the same kinetic inhibition model and with appropriate statistical significance (Figure 6E; Supplementary Figure S2B). These values are in concordance with the productive binding of the Sm-ζ into the PdF_1_F_O_-ATPase with lower affinity than its homologous Pd-ζ, as expected from a heterologous reconstitution. In a reciprocal experiment, we assayed the putative heterologous inhibition of the Rhizobiales F_1_-ATPases (SmF_1_ and ReF_1_) with the recombinant Pd-ζ. After the custom reconstitution of Pd-ζ in the presence of MgATP into SmF_1_ and ReF_1_ ATPases, we could not observe any inhibitory function of the Pd-ζ whatsoever. Instead of repeating these experiments with negative results, we used a considerable excess of recombinant Pd-ζ, ≥100μg to confirm that there were no inhibitory effects of Pd-ζ on SmF_1_ and ReF_1_ ATPases (see Figures 6B,C). These results indicate that the structural differences (Figures 6F, 8A, C) between Pd-ζ and Sm-ζ (and/or PdF_1_ and SmF_1_, see Supplementary Material) make Sm-ζ unable to inhibit its own SmF_1_-ATPase and Pd-ζ non-compatible to inhibit the Rhizobiales SmF_1_ and ReF_1_ ATPases. However, Sm-ζ can inhibit heterologously the PdF_1_- and PdF_1_F_O_-ATPases. Thus, the PdF_1_ and PdF_1_F_O_ nanomotors are somehow able to induce the inhibitory N-terminal extended-α-helical conformer of Sm-ζ but not their own SmF_1_-ATPase.

**FIGURE 7:**
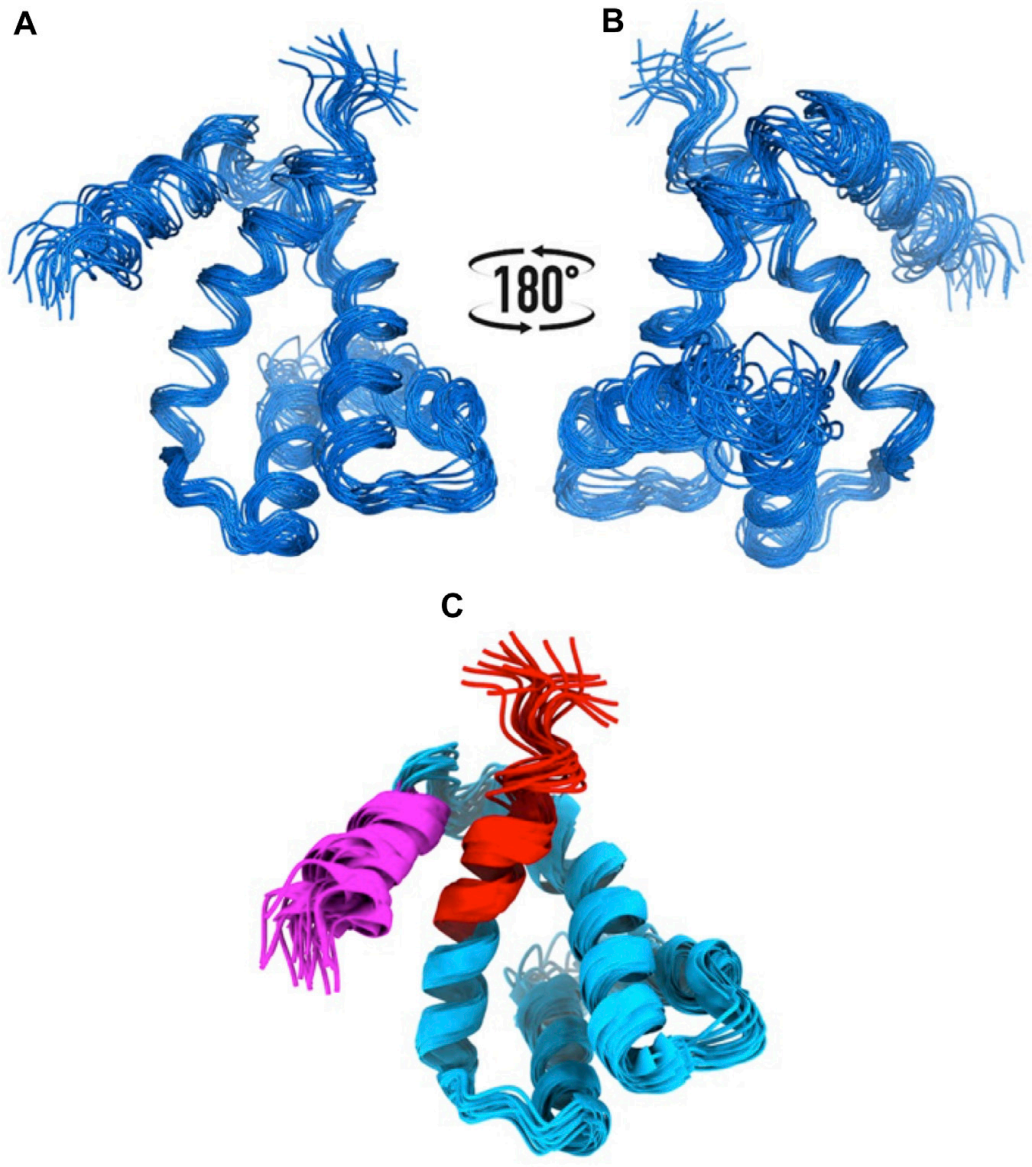
NMR structure of the Sm-ζ. **(A), (B)** Bundle of 20 superimposed conformers, where only backbone atoms are shown. **(C)** Ribbon representation of the 20 conformers; the N-terminus is colored in magenta, and the C-terminus is colored in red.

**FIGURE 8:**
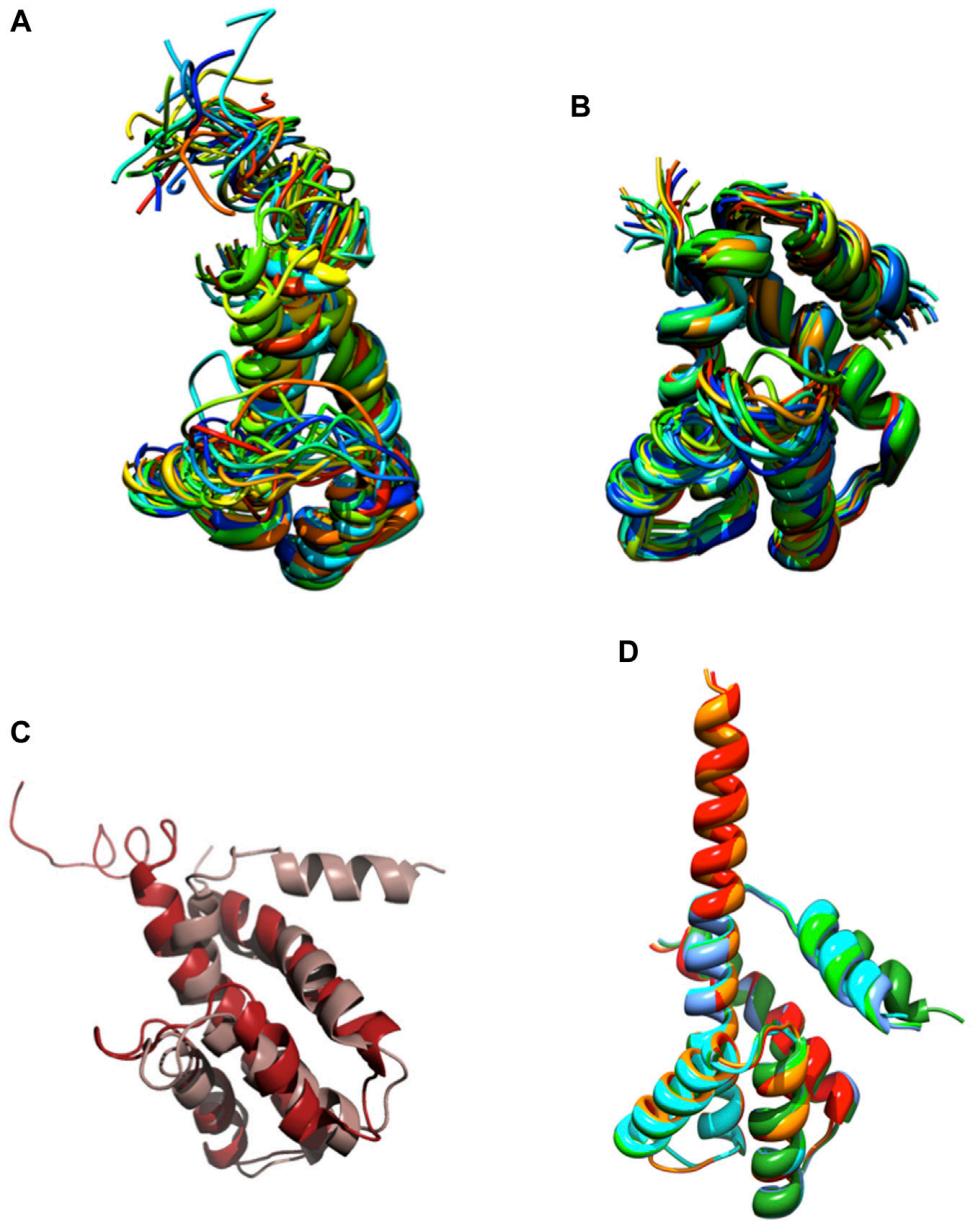
NMR and AF structures of Pd-ζ and Sm-ζ. **(A)** The 20 conformers originally resolved by NMR of the Pd-ζ subunit in rainbow ribbons with the N-terminus showed as an IDPr highly mobile region. **(B)** The 20 NMR conformers of the Sm-ζ showed in rainbow ribbons, with its N-terminus folded as a first α-helix of a 5-α-helix globular bundle, twisted and interacting with the fifth α-helix. **(C)** Superposition of the Pd-ζ (PDB_id 2LL0, red) and Sm-ζ (PDB_id 7VKV, light brown) showing the opposing orientations of their N-termini. **(D)** Structures of AF models of Sm-ζ subunits identical to our NMR-resolved ζ (red), nearly identical (orange), and more divergent (dark and light green, cyan, and blue) to the NMR-resolved Sm-ζ (PDB_id 7VKV) shown in **(B)**. The closest AF models to our NMR-resolved SM-ζ (red and orange) show the inhibitory N-terminal extended Sm-ζ conformation, whereas the less-similar Sm-ζ structures (light green, dark green, blue, and cyan) show the non-inhibitory 5-α-helical globular bundle conformation. See the text and Supplementary Figure S6; Supplementary Table S2 for further details.

We knew, at this point, that Sm-ζ exhibits an α-helical content similar to that of Pd-ζ, according to the CD results (Figure 5C), and therefore, the overall folding of both ζ subunits (Sm-ζ and Pd-ζ) should be similar. However, to analyze more deeply the structure of Sm-ζ and look for structural differences between Pd-ζ and Sm-ζ that could explain the functional differences of both ζ subunits, we resolved the NMR structure of Sm-ζ in a similar fashion to our previous solution structure of Pd-ζ ((Zarco-Zavala et al., 2013; Serrano et al., 2014; Zarco-Zavala et al., 2014) and PDB_id 2LL0). The structure resolved, showing the 20 most representative conformers of Sm-ζ exhibited a similar globular folding as Pd-ζ, as expected from the CD results (Figure 5C). However, the Sm-ζ N-terminus showed a radically different structure compared with Pd-ζ. In the case of Pd-ζ, we consistently observed the inhibitory N-terminus as an intrinsically disordered protein region (IDPr) with high mobility in solution, which shifts to an extended N-terminal α-helix after its productive binding to the INGECORE of the α_DP_β_DP_γ interface of the PdF_1_ (Garcia-Trejo et al., 2016) and PdF_1_F_O_ ATPases (Morales-Rios et al., 2015). In the case of Sm-ζ, its N-terminus is not disordered in solution at all, it forms a well-folded α-helix, but it is not extended; instead, it bends toward the C-terminal α-helix so that the final conformer of Sm-ζ in solution is a globular and compact 5-α-helix bundle (Figures 7, 8), in contrast to the 4-α-helix bundle of Pd-ζ (PDB_id 2LL0 and Figure 8A). Figure 7A, B shows two side views of the NMR structure of Sm-ζ (PDB_id code 7VKV), with the N- and C-termini of Sm-ζ viewed from the “back” and “front” of the protein, respectively. For more clarity, the N- and C-termini of Sm-ζ are colored violet and red, respectively, in Figure 7C, with the *red* C-terminus viewed at the front and the *violet* N-terminus protruding from the back, respectively (Figure 7C). Here, the Sm-ζ has the same orientation and view as in Figure 7A. As can be seen, there is no IDPr at the N-terminus of Sm-ζ as in the case of Pd-ζ (PDB_id 2LL0 and Figure 8A). One of the first questions that emerge from this structure is whether it is consistent with the content of α-helix determined in solution by CD (Figure 5C). Accordingly, the content of α-helix of Sm-ζ was calculated as the number of aa´s in α-helical structure from the most representative conformer of the 20 resolved by NMR (PDB_id 7VKV) with the STRIDE site (http://webclu.bio.wzw.tum.de/cgi-bin/stride/stridecgi.py), giving an α-helical content of 72.1%. This is in good agreement with the 75.7% of α-helix as obtained by our CD experiments (Figure 5C). As an internal control, the α-helical content of the most representative control Pd-ζ conformer was also calculated from the NMR PDB_id 2LL0, and we obtained a value of 60.5%, which is similar but lower to the 74.8% of α-helical content calculated from the CD spectra (Figure 5C). The slight discrepancies of smaller α-helical content obtained by NMR in both Sm-ζ and Pd-ζ may be due to the differences in the media used for both determinations, particularly in the slightly acidic pH of the media necessary for the NMR experiments (pH ≈ 6.0–6.8), whereas the CD spectra of both Sm-ζ and Pd-ζ were carried out at more basic pH (pH 8.0). We have shown that the more effective inhibitory activity of Pd-ζ is obtained at more alkaline (pH 8.0) than at more acidic pH (6.0) (Morales-Rios et al., 2010; Morales-Rios, 2011; Zarco-Zavala et al., 2014; Mendoza-Hoffmann et al., 2018; Zarco-Zavala et al., 2018); therefore, it seems consistent to see a slightly higher α-helical content in both Sm-ζ and Pd-ζ by CD at pH 8.0 than by NMR at pH ≈ 6.0–6.8. In summary, the CD and NMR structures of Sm-ζ complement each other very well and show that the solution structure of Sm-ζ is a globular 5-α-helical bundle devoid of the disordered N-terminal IDPr, i.e., in radical contrast to the IDPr of Pd-ζ, although both structures may present similar α-helical contents at pH 8.0.

The ordered and α-helical folded structure of Sm-ζ explains why it is unable to inhibit its own SmF_1_-ATPase (Figure 6A). It seems that the activation energy for the transition from the 5-α-helical bundle to the extended N-terminal α-helical inhibitory conformation is a thermodynamic and/or kinetic barrier that is too high and cannot be properly triggered by the SmF_1_-ATPase. Looking for primary structure differences between the Sm-ζ and the Pd-ζ, we found significant changes in the first 14 N-terminal residues, which, as we demonstrated, harbor the inhibitory domain of Pd-ζ (Zarco-Zavala et al., 2014) (see the red box in Figure 6F). These changes, together with a few others in the rest of Sm-ζ and possibly in the INGECORE *α*_DP_ *β*_DP_γ interface of the SmF_1_ (Mendoza-Hoffmann, 2018) (see Supplementary Figure S7), should explain the inability of the SmF_1_-ATPase to trigger the inhibitory transition from the compact 5-α-helical globular Sm-ζ conformer (Figures 7, 8) to the extended N-terminal inhibitory form of Sm-ζ (Figure 8D). In summary, the data show that Sm-ζ has a compact non-inhibitory 5-α-helical globular conformation that likely leaves the isolated SmF_1_-ATPase essentially devoid of bound Sm-ζ as detected by SmF_1_ SDS-PAGEs and anti-ζ Western blots (Figure 4).

An intriguing question is why the Sm-ζ is unable to inhibit its own SmF_1_-ATPase but still able to inhibit heterologously the PdF_1_-ATPase. This observation indicates that the PdF_1_-ATPase is somehow able to trigger the transition from the compact non-inhibitory (5-α-helical bundle) conformer of Sm-ζ into the extended N-terminal α-helical inhibitory conformer of Sm-ζ. We looked for structural insights that might explain this heterologous inhibitory effect of Sm-ζ, so we looked for the putative Sm-ζ structures modeled in AF. We found six structures of Sm-ζ predicted by the AF database, but these structures had different sequences and structures, originating from different *S. meliloti* strains (see Supplementary Table S3). When we compare the sequence of these AF models with the sequence of the Sm-ζ structure that we resolved by NMR (PDB_id 7VKV), they have the following identities: 100%, 99%, 58.65%, 57.28%, 60.19%, and 60.19% (Supplementary Table S3). We compared these AF models with each other (Figure 8D) and with our NMR structure (Supplementary Figure S6). Interestingly, when AF used Sm-ζ sequences with 100% and 99% identity with our Sm-ζ, it predicted a different structure (Figure 8D). This AF structure shows the extended N-terminal α-helical inhibitory conformation (Figures 8D, Supplementary Figures S6A–D), similar to the AF models of *C. sphaeroides* (Figure 2D) or Rhodospirillaceae *bacterium* (Figure 3D) and the Pd-ζ subunit bound to its inhibitory INGECORE site in the PdF_1_-ATPase (Mendoza-Hoffmann et al., 2018; Zarco-Zavala et al., 2018). The other AF models with ∼ 60% identity to our Sm-ζ (PDB_id 7VKV) showed a compact non-inhibitory 5-α-helical conformation similar to our resolved compact non-inhibitory 5-α-helical NMR structure of the PDB_id 7VKV (see Figure 8D and Supplementary Figures 6SE–L). Although, in general, the N-termini of the AF ζ models have the lowest confidence in structure prediction (see, for instance, the AF model Sm-ζ structures in Supplementary Table S3), we consider that these N-terminal α-helical extended and 5-α-helical compact conformations of Sm-ζ predicted by AF may represent the inhibitory and non-inhibitory conformers that Sm-ζ could adopt either isolated in solution or bound productively to inhibit a compatible F_1_-ATPase. In solution, the compact 5-α-helical globular folded conformer is more enriched according to our CD (Figure 5C) and NMR (Figures 7, 8B) results. However, it seems that the inhibitory conformer predicted by the AF models shows that the Sm-ζ, although being non-inhibitory on its own SmF_1_-ATPase, preserves within its sequence the inhibitory potential to adopt the N-terminal α-helical extended inhibitory conformation (Figure 8D; Supplementary Figure S6A). This is in full agreement with our observation that the Sm-ζ is somehow able to inhibit, after heterologous reconstitution, the PdF_1_ and PdF_1_F_O_-ATPases of *P. denitrificans*. How the ATP synthase of *P. denitrificans* is able to induce the transition to the N-terminal extended inhibitory conformation of Sm-ζ will be a question addressed in the discussion.

### The absence of the αPATPsζ gene and the ζ subunit in Rickettsiales and other parasitic or symbiotic α-proteobacteria

**FIGURE 9:**
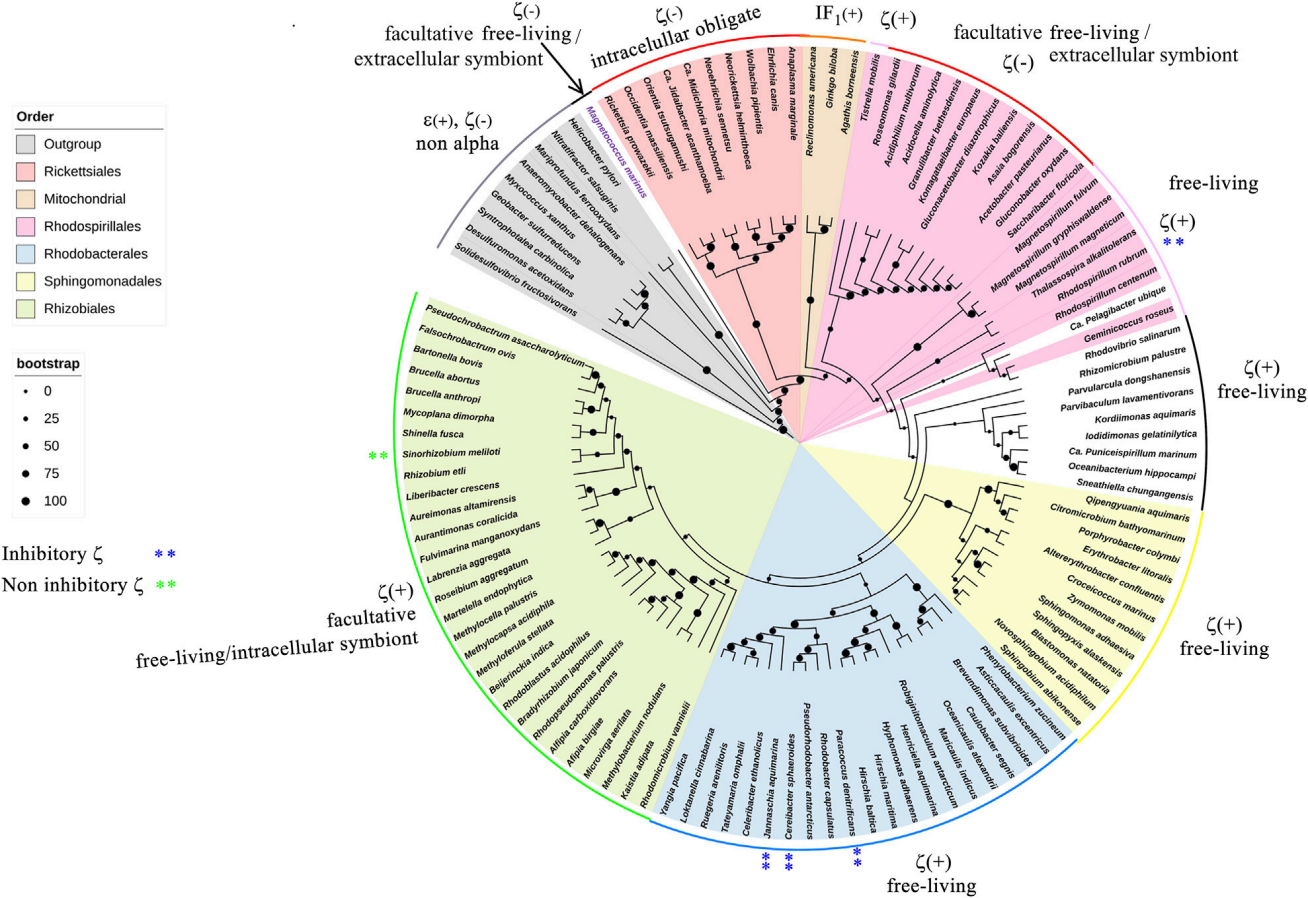
16S rRNA phylogenetic three of α-proteobacteria and mitochondria. The 16S phylogeny was carried out by taking 114 sequences of ribosomal 16S from102 different species of different orders that represent the α-proteobacteria class. These orders were Rhodobacterales, Rhizobiales, Rhodospirillales, Sphingomonadales, Caulobacterales, Sneathielalles, Parvularculales, Pelagibacterales, and Kordimonadales, among others. Additionally, nine 16S sequences belonging to ε- or δ-proteobacteria were used, and three homologous mitochondrial sequences were used. Most sequences were retrieved from the NCBI nucleotide database, and only the mitochondrial sequences were retrieved from the SILVA database (https://www.arb-silva.de/). The alignment and phylogeny were carried out as detailed in the work of Mendoza-Hoffmann et al. (2022).

**FIGURE 10:**
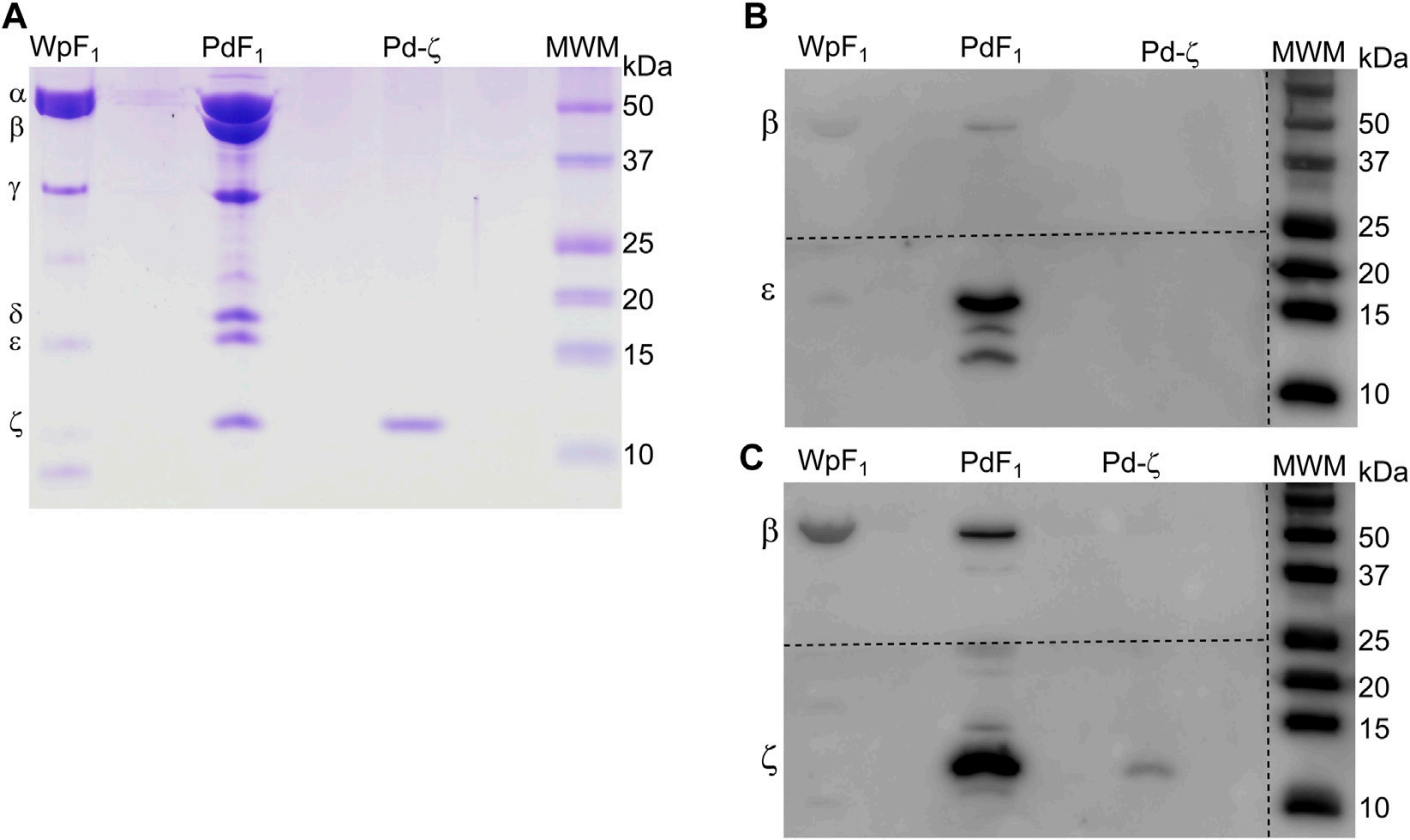
Confirmation of the absence of the ζ subunit in the F_1_ of *W. pipientis*. **(A)** Coomassie-stained SDS-PAGE of the purification of *W. pipientis* F_1_ (F_1_ Wp). **(B)** WB anti-ε of the Wp F_1_ complex. **(C)** WB anti-ζ of the WpF_1_ complex. The anti-ζ WB membrane was developed first. Then, the antibodies were removed (stripping), and the membrane was exposed to the anti-ε antibody and revealed again **(B)**. The membrane was cut to incubate with the different antibodies, as shown by the discontinuous lines.

Once we found that ζ has lost its inhibitory potency in some symbiotic α-proteobacteria, it seemed possible that the gene could have been lost in strictly parasitic or symbiotic α-proteobacteria, given that symbionts and parasites may obtain nutrients or ATP directly from their hosts. For instance, it is well known that Rickettsiales have an inward ATP membrane transporter that consumes the host´s cellular ATP (Andersson et al., 1998). This would make the ζ subunit totally dispensable given that the Rickettsiales ATP synthase is not so urged to synthesize ATP, but it could still hydrolyze it as a proton pump to keep the transmembrane proton gradient to fulfill the chemiosmotic energy requirements of the Rickettsiales. In the search of the αPATPsζ gene, we found it absent in most of the Rickettsiales and in some other α-proteobacteria as in the Rhodospirillales order (see Figure 9). We also found the latter to be, in all cases, facultative or free-living extracellular symbionts. As described previously (Mendoza-Hoffmann et al., 2022), we found that the αPATPsζ gene is absent in the family Holosporaceae from the order Holosporales, the family Acetobacteraceae from the order Rhodospirillales, and most of the species in the order Rickettsiales. This lack of Rickettsiales’ ζ may have important implications in the search for the identity of the mitochondrial endosymbiont that evolved into the present mitochondria (Mendoza-Hoffmann et al., 2022). Therefore, in order to prevent the possibility of the αPATPsζ gene being present but skipped or missed in our bioinformatics search in Rickettsiales, we confirmed biochemically that the ζ subunit is totally absent in one species of these Rickettsiales. We isolated the F_1_-ATPase from *Wolbachia pipientis* (WpF_1_), grown as an intracellular symbiotic host in human erythrocytes; since the latter lack mitochondria, this ensured the absence of putative contaminant mitochondrial F-ATPase (mtF-ATPase) present, for instance, in yeast that was used before as the *W. pipientis* host (Uribe-Alvarez et al., 2019). As expected, from the absence of the αPATPsζ gene in *W. pipientis*, we found by WpF_1_ purification and anti-ζ Western blot that Wp-ζ is totally absent in the WpF_1_-ATPase (Figure 10C), where we confirmed the presence of the α, β, γ, and ε subunits (Figures 10A, B). This experiment demonstrates biochemically the absence of both the αPATPsζ gene and the ζ subunit protein in the Rickettsiales order of α-proteobacteria. As discussed in the following section, the absence of the ζ subunit in these α-proteobacteria orders may have important implications in the evolution of mitochondria from α-proteobacteria, as suggested by Sagan (1967), Margulis and Chapman (1998), Andersson et al. (1998), Gray et al. (1999), Archibald (2015), and Ku et al. (2015).

### MD simulation of the transition from the N-terminal disordered to the ordered inhibitory extended *α*-helical conformer in Pd-*ζ*

**FIGURE 11:**
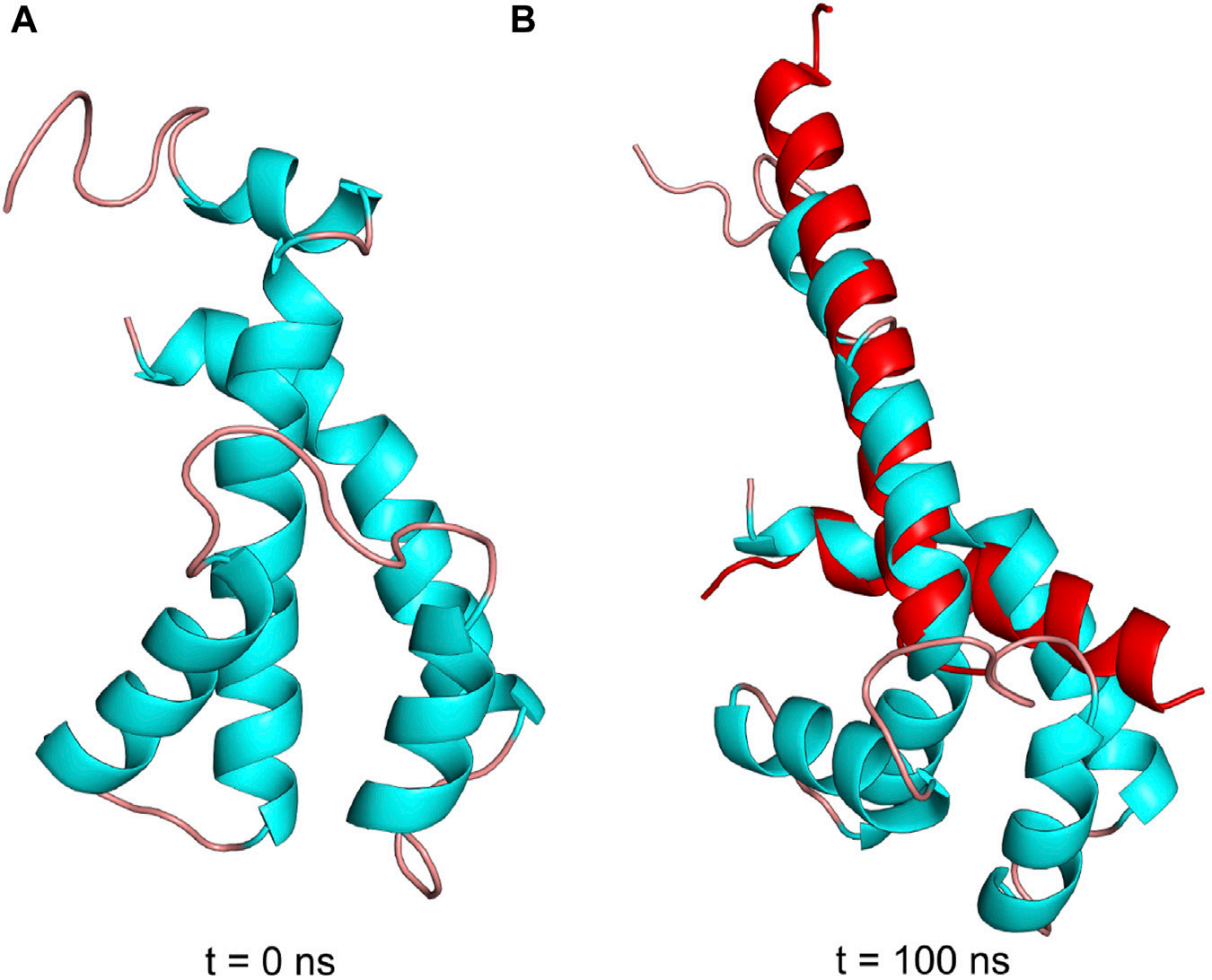
Pdζ structures during simulation at pH 8. **(A)** Initial structure in the MD simulation at constant pH (pHMD). **(B)** Structure after 100 ns of MD simulation. In red is the structure of the N- and C-termini of Pd-ζ obtained by crystallography (PDB_id 5DN6). As can be seen, the pHMD simulation predicts the spontaneous ordering and formation of the extended N-terminal inhibitory α-helix of Pd-ζ, very similar to the inhibitory N-terminal extended conformation of Pd-ζ bound to its inhibitory site in the native PdF_1_F_O_-ζ complex (PDB_id 5DN6, red). See also Supplementary Movie S1; Supplementary Figures S8A–D for details.

The structural details that induce the inhibitory transition from the N-terminal disordered to the ordered inhibitory α-helical conformer of Pd-ζ upon productive binding to PdF_1_ are far from being resolved here. However, in an attempt *in silico* to make evident that the Pd-ζ could undergo this transition from non-inhibitory and intrinsically disordered structure (PDB_id 2LL0) to the inhibitory N-terminal extended α-helical structure (PDB_id 5DN6), we were able to simulate this disordered to α-helical extended inhibitory transition in the Pd-ζ subunit by MD analysis carried out at constant pH 8.0, suggesting that this transition could be reached spontaneously in solution (Figure 11 and Supplementary Video S1). With this, we make evident that this inhibitory conformation was obtained, although at low statistical frequency, given that it was observed in only one of the MD analyses carried out in triplicate (see Supplementary Figure S8 and Supplementary Movie S1). Taken together with the extended inhibitory conformers predicted by AF, we show that this disordered to the α-helical extended inhibitory transition of ζ can be induced by the proper α-proteobacterial ATP synthase, preferably the one from *P. denitrificans*. Alternatively, it may also take place spontaneously, although perhaps sporadically, in the Pd-ζ isolated in solution at the optimal inhibitory pH of 8.0. Furthermore, we also carried out a similar MD analysis at constant pH 8.0 of the Sm-ζ NMR structure (PDB_id 7VKV) to compare it with that of Pd-ζ. The MD results clearly showed that the Sm-ζ was unable to undergo the transition from the compact non-inhibitory 5-α-helical bundle conformer (Figures 7, 8B) to the inhibitory N-terminal extended α-helical conformer (Figures 8D and Supplementary Figure S6A), at least in the triplicate MD simulations carried out. A representative video of the three MD replications of Sm-ζ can be seen in Supplementary Video S2, and the trajectories of the three replications are shown in Supplementary Figures S8A–D. In summary, the *in silico* MD results are in concordance with the inability of Sm-ζ to inhibit its own SmF_1_-ATPase (Figure 6A) since it shows higher thermodynamic or kinetic requirements, and, thus, a lower probability than Pd-ζ, to achieve the transition from its non-inhibitory 5-α-helix bundle compact conformation to the inhibitory extended α-helical conformer. This inhibitory transition cannot be triggered by its own SmF_1_-ATPase, but it can be somehow induced by the PdF_1_ and PdF_1_F_O_ complexes.

## Discussion

The results presented here open a new window by showing that, in general, ζ evolved in concordance with its functional and bioenergetic role in the different bacterial families. This is because we found that the inhibitory capacity of ζ is preserved in free-living, more environmentally challenged α-proteobacteria, reduced or lost in some symbiotic α-proteobacteria, and completely lost in some symbiotic or in most strictly parasitic Rickettsiales α-proteobacteria. The latter α-proteobacteria are less challenged by environmental changes and may obtain nutrients and/or ATP from their hosts, thus making the ζ subunit dispensable in symbiotic or parasitic α-proteobacteria. Exceptions to this evolutionary hypothesis of the ζ subunit may appear in nature, but more functional and evolutionary studies are needed to confirm this hypothesis and to show the exceptions on this trend of preserving the ζ inhibitory function in free-living α-proteobacteria and losing its function or the ζ gene in symbiotic and parasitic α-proteobacteria. The latter is coincidentally closely related to the endosymbiotic origin of mitochondria.

In addition, we resolved and correlated the NMR structure of the Sm-ζ with its lack of inhibitory function, which is in concordance with its compact 5-α-helical non-inhibitory conformation (Figures 7, 8). We also found by AF modeling that Sm-ζ may acquire the extended N-terminal α-helical inhibitory conformation as confirmed by heterologous inhibition of the PdF_1_ and PdF_1_F_O_ ATPases (Figures 6E, 8D). We still need to explain the heterologous, but not homologous, inhibitory function of Sm-ζ on the PdF_1_ and PdF_1_F_O_ ATPases. In this regard, we have recently shown that in hybrid F_1_-ATPases constructed with at least one PdF_1_-ATPase subunit (either Pd-α, Pd-β, or Pd-γ), these PdF_1_-ATPase subunits induce the functional conformations of F_1_ subunits from other species (i.e., bovine or thermophilic bacterial subunits) to rotate according to the newly described 3° × 120° rotation unique of the PdF_1_-ATPase and not according to the multiple rotary stepping in the other bacterial or mitochondrial F_1_-ATPases (Zarco-Zavala et al., 2020). This indicates that the PdF_1_ individual subunits have the potential to induce other heterologous F_1_ subunits to adapt to their structure and catalytic rotary mechanism; thus, the PdF_1_ subunits work as robust dominant subunits (Watanabe et al., 2023). It seems, therefore, suitable to suggest that the PdF_1_-ATPase, but not the SmF_1_, is similarly able to induce the structural transition from the compact 5-α-helical non-inhibitory conformer of Sm-ζ (Figures 7, 8B), into its inhibitory N-terminal extended α-helical conformation (Figures 8D; Supplementary Figure S6A) to produce the observed PdF_1_ or PdF_1_F_O_-ATPase inhibition.

MD analyses have been previously helpful in analyzing the inhibitory and regulatory mechanisms of the ε subunit from non-α-proteobacteria (Krah and Takada, 2016; Krah et al., 2021; Krah et al., 2023) and the mitochondrial IF_1_ (Domínguez-Ramírez et al., 2006), describing differences in regulatory ATP binding in ε and a hinge separating the inhibitory and anchoring domains of IF_1_. Thus, we carried out MD analyses of the ζ subunit to shed light on the inhibitory and regulatory mechanisms of the ζ subunit. The MD analyses carried out here showed clearly that the inhibitory Pd-ζ subunit may experience, although sporadically, since it was observed in one of the 3 MD replications, a spontaneous transition from the N-terminus disordered conformation to the N-terminus extended α-helical conformation mimicking the inhibitory conformer of Pd-ζ bound productively to the PdF_1_ (Garcia-Trejo et al., 2016) or PdF_1_F_O_ complexes (Morales-Rios et al., 2015). However, in its three MD replications, the non-inhibitory Sm-ζ subunit was unable to experience a similar transition from the ordered and folded 5-α-helical bundle to the N-terminal extended inhibitory α-helical conformer of Sm-ζ (see Figure 11 and Supplementary Figure S8 and Supplementary Videos 1, 2). This is in concordance with the lack of inhibitory function of Sm-ζ on its own SmF_1_-ATPase and indicates that a larger energetic barrier exists in Sm-ζ to achieve the transition from the compact non-inhibitory conformation to its N-terminal α-helical extended inhibitory conformer. Somehow, the PdF_1_-ATPase, but not the SmF_1_-ATPase, is able to overcome this energetic barrier due to its tendency to work as a dominant PdF_1_-ATPase to induce functional conformations on the reconstituted ATP synthases’ heterologous subunits from other species, adapted to the PdF_1_-ATPase subunits (Watanabe et al., 2023), thus taking the Sm-ζ to the N-terminal α-helical extended inhibitory conformer.

**FIGURE 12:**
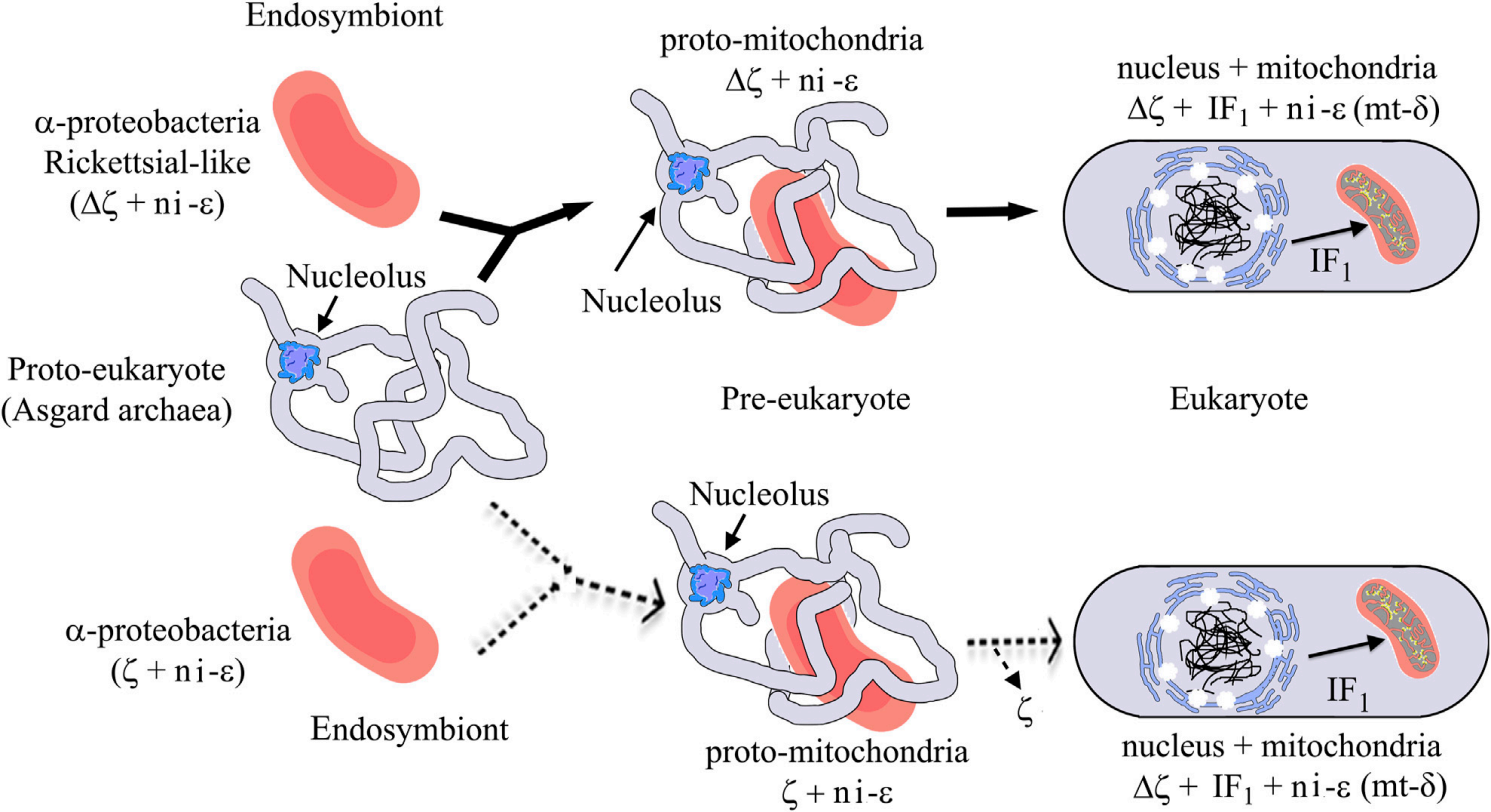
Mitochondrial endosymbiosis with an α-proteobacterial pre-endosymbiont having or lacking the ζ gene. The upper panel (black arrows) shows the most likely mitochondrial endosymbiotic scenario, with ζ non-homologous to IF_1_ and both proteins inhibiting the bacterial or mitochondrial ATP synthases by convergent evolution and with a Rickettsial-like endosymbiont lacking the αPATPsζ gene (Δζ), the latter invading intracellularly a protoeukaryote (Asgard archea), probably already having a nucleolus (Islas-Morales et al., 2023). This evolved from being an intracellular symbiont or parasite into a protomitochondria devoid of ζ and having a non-inhibitory ε subunit (ni-ε) in its F-ATP synthase that will become the mitochondrial mtATP synthase δ subunit (mt-δ). This eventually transforms into mitochondria in an enucleated eukaryote. In mitochondria, IF_1_ emerged not only to inhibit preferably the F_1_F_O_-ATPase activity but also to stabilize the mitochondrial F-ATP synthase dimers and further oligomers that give its shape to the cristae of the mitochondrial inner membrane (see yellow spots of F-ATP synthases in the mitochondrial cristae). The lower part (dashed light arrows) shows a less-likely scenario where an α-proteobacterial endosymbiont having a non-inhibitory ζ and a ni-ε became a protomitochondria in the pre-eukaryote; here, the ni-ζ vanished and IF_1_ emerged independently in the mitochondria of the enucleated eukaryote. See the text for further details. Figures are adapted from the work of Imachi et al. (2020) and Mendoza-Hoffmann et al. (2022).

Our evolutionary results might have important implications in the endosymbiotic evolution from α-proteobacteria to mitochondria. In the case of the origin of mitochondria, it is strongly suggestive that the origin of mitochondria, as shown by rRNA evolution (Figure 9), indicates that the parasitic or symbiotic α-proteobacteria lacking the ζ subunit are surrounded very closely by the origin of mitochondria (Figure 9), with the latter most likely evolving from these α-proteobacteria (John and Whatley, 1975a; John and Whatley, 1975b; Andersson et al., 1998; Gray et al., 1999; Archibald, 2015; Ku et al., 2015) (although some other proposals outside the α-proteobacteria have also emerged (Martijn et al., 2018; Cevallos and Degli Esposti, 2022)). Thus, we suggest two scenarios, of which the most likely is the one in which mitochondria evolved from a pre-mitochondria originating from an endosymbiotic event involving a protoeukaryote (likely Asgard archaea, which probably already had a nucleolus, as shown recently (Islas-Morales et al., 2023)) and a Rickettsiales-like α-proteobacteria endosymbiont already lacking ζ (Δζ) and having a non-inhibitory ε subunit that eventually became the mitochondrial non-inhibitory δ subunit of the mitochondrial F-ATPase (mtATPase) (Mendoza-Hoffmann et al., 2022) (Figure 12). This settled the evolutionary pressure for the independent emergence by *evolutionary convergence* of the mitochondrial IF_1_ as the endogenous inhibitor of the mtATPase, which is not homologous to the α-proteobacterial ζ subunit (Mendoza-Hoffmann et al., 2022). IF_1_ eventually also promoted the mtATPase dimerization, oligomerization, and mitochondrial cristae formation, as we demonstrated previously (Minauro-Sanmiguel et al., 2005; Garcia et al., 2006). In a less-likely scenario, the protomitochondria might have evolved from a protoeukaryote and an endosymbiotic α-proteobacteria having a non-inhibitory ζ subunit that was eventually lost during the transition from protomitochondria to modern mitochondria, with the emergence of IF_1_ by *convergent evolution* and the concomitant formation of mitochondrial cristae (Figure 12, bottom panel). We propose this scheme based on the mutually exclusive presence of ζ or IF_1_ in α-proteobacteria and mitochondria, respectively, and their non-homologous character (see also the work of Mendoza-Hoffmann et al. (2022)).

## Data Availability

The original contributions presented in the study are included in the article/Supplementary Material, further inquiries can be directed to the corresponding authors.
