# Supplementary material for: Inhibitory to non-inhibitory evolution of the ζ subunit of the F1FO-ATPase of Paracoccus denitrificans and α-proteobacteria as related to mitochondrial endosymbiosis
Source: Front Mol Biosci. 2023 Aug 17;10:1184200. doi: 10.3389/fmolb.2023.1184200 (PMC10469736; doi:10.3389/fmolb.2023.1184200)
Supplement: Supplementary file 1 [file DataSheet1.pdf]

Preparative alignments and conservation analyses of the ζ and ε subunits of the ATP synthases of α-proteobacteria.

In order to study the evolution and the function of the ζ subunit across the α-proteobacteria class, we started with the bioinformatic analysis to have a comprehensive view of the conservation of the ζ subunit along α-proteobacteria. To accomplish this, we obtained updated and more complete sequences than in previous alignments [11], confirming that the ζ subunit is essentially a protein family (DUF 1476) belonging to the α-proteobacteria class. We previously named the ζ gene as the "αPATPsζ" gene (α-proteobacterial F-ATP synthase ζ gene) [27]. We do not call it "ATPζ" to avoid confusion since this gene is not part of neither of the two ATP (previously *UNC*) operons of α-proteobacteria. We found some "αPATPsζ" gene hits outside the α-proteobacteria class that are mostly truncated pseudogenes, likely present in other bacterial classes due to horizontal transfer. The ζ subunit is similar to some enzymes, such as those identified as aldolases or enzymes involved in purine biosynthesis that could have acquired a ζ-like domain in α-proteobacteria and other bacterial classes. Importantly, we also confirmed, as reviewed before [27], that the ζ gene is absent in eukaryotic organisms, except for two unicellular eukaryotes that are either commensals or symbionts of other more complex eukaryotes, thus coexisting in close proximity with several α-proteobacteria, from which they could have acquired the αPATPsζ gene by horizontal transfer [27]. This is important because it also confirms that ζ is not a homologous predecessor of the mitochondrial IF_1 but an analogous inhibitory protein of the α-protobacterial F-ATPase [27]. We can therefore conclude that the ζ subunit or "αPATPsζ" gene as an isolated F-ATPase inhibitor, is essentially exclusive of the α-proteobacteria class.

We also confirmed the previously observed strict conservation of the functional N-inhibitory domain of the ζ subunit family and the gradually increasing divergence of the protein family from the inhibitory N-terminus towards the C-terminus [11; 27]. This is clearly illustrated by the CONSURF analysis showing the most preserved regions (cherry-red sections) at the N-terminus of the ζ family and in some central

residues of the α-helix one and the α-Helix2-Loop2-α-Helix 3 (αH2L2H3) domain (Figure S1A). Furthermore, the higher conservation of the very N-terminus of the ζ subunit across α-proteobacteria coincides with the functional inhibitory domain also found within these first 14 residues of the ζ subunit of *P. denitrificans*, or the first 19 residues of the ζ subunit of *Jannaschia sp*. [11]. Some other conserved residues (shades of cherry-red) also found in the αH2L2H3 domain are likely crucial for interactions of the ζ globular domain with the α/β subunits working as an anchoring or ligand binding domain. The most variable side of the ζ subunits is the C-terminal α-Helix 4, which does not seem to have a crucial role, but perhaps adds structural stability to the ζ protein. This conservation of functional domains of ζ is in agreement with our modeled structure of the full PdF1FO-ζ complex showing that the globular domain of ζ interacts mainly with the C-terminus domain of the β_{DP} subunit [12].

On the other hand, we also verified by CONSURF the conservation of the non-inhibitory ε-subunit of the ATP synthase of α-proteobacteria. As reviewed before [6; 27], we confirmed that its N-terminus side, i.e., the c-subunit anchoring domain, is well conserved (shades of cherry-red Figure S1B) in analogy with the N-terminus of ζ. However, the C-termini of α-proteobacterial ε subunits are very variable, in several cases even truncated, thus losing the F-ATPase inhibitory function of this domain, as well as the regulatory ATP binding cassette (I(L)XXRA) [6; 27]. Furthermore, we also found that the non-inhibitory α-proteobacterial ε is an evolutive predecessor of the non-inhibitory mitochondrial ATP synthase´s δ subunit [27], in concordance with the endosymbiotic theory of Lynn Margulis [28], strongly suggesting the endosymbiotic origin of mitochondria within the α-proteobacteria class [29].

Others have proposed that besides MgADP and ζ, Pd-ε also has a regulatory role on the PdF_1F_O ATP synthase ([13; 14]). However, in their recent study [13], instead of showing that their Pd-$\varepsilon^{\Delta CT}$ truncated mutants activate significantly the PdF_1F_O-ATPase, it clearly demonstrates that the only single or double mutants where a significantly higher PdF_1F_O-ATPase activation occurs (from ≈0.02-0.04 nmol/(min.*mg.pt.)) to ≈ 400-750 nmol/(min.*mg.pt.), see Figure 4B of ref. [13]), are

exclusively those where the ζ subunit was removed genetically and not those where only Pd-ε was truncated [13]; however, this clear result was not discussed in that work. Recently, with another similar C-terminal Pd-$\varepsilon^{\Delta CT}$ truncated mutant, we had confirmed that neither the Pd-ε^{WT}, nor the Pd-$\varepsilon^{\Delta CT}$ truncated mutant exert any inhibition whatsoever on the PdF_1-ATPase activity [16],. We also demonstrated before biochemically the total absence of any direct or indirect inhibitory effect of Pd-ε by its reconstitution on the PdF_1-ATPase [11], which was confirmed more recently by phylogenetic analyses (ref. [16]). Our present Supplementary Material (see above and figure S1B) revisits bioinformatically the lack of inhibitory function of the α-protobacterial ε in other α-protobacteria besides *P. denitrificans*, so taken together ,several data demonstrate that the ε subunit from α-proteobacteria has lost its inhibitory and ATP binding functions, and this has been reviewed before [6; 27]. It seems therefore suitable to conclude that the biochemical, knockout mutant, and evolutive bioinformatic phylogenetic evidence revised before and now, confirms that in general, the main inhibitory role to control or prevent the futile F_1F_O-ATPase activity was completely lost by the α-proteobacterial ε and was fully acquired by the ζ subunit [6; 27], although perhaps some exceptions to this trend may eventually occur in α-proteobacteria.

Figure S1

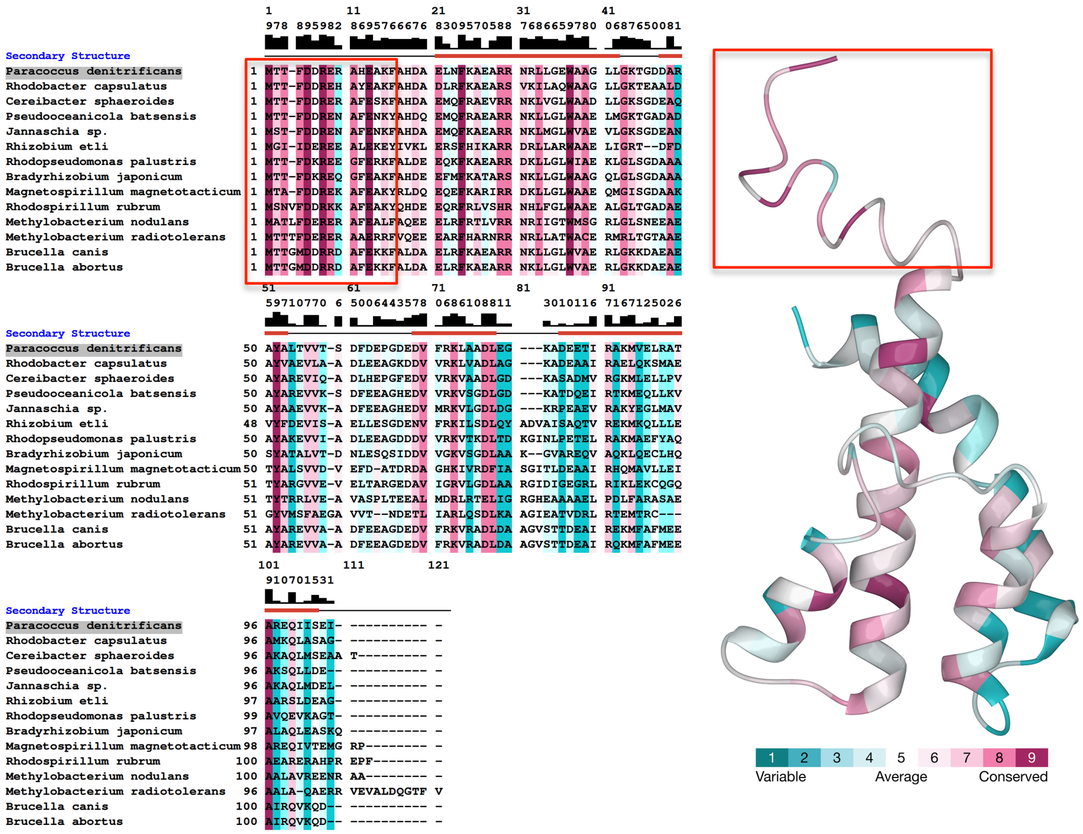
Secondary Structure
Paracoccus denitrificans
Rhodobacter capsulatus
Cereibacter sphaeroides
Pseudooceanicola batsensis
Jannaschia sp.
Rhizobium etli
Rhodopseudomonas palustris
Bradyrhizobium japonicum
Magnetospirillum magnetotacticum
Rhodospirillum rubrum
Methylobacterium nodulans
Methylobacterium radiotolerans
Brucella canis
Brucella abortus
1 2 3 4 5 6 7 8 9
Variable
Average
Conserved

B

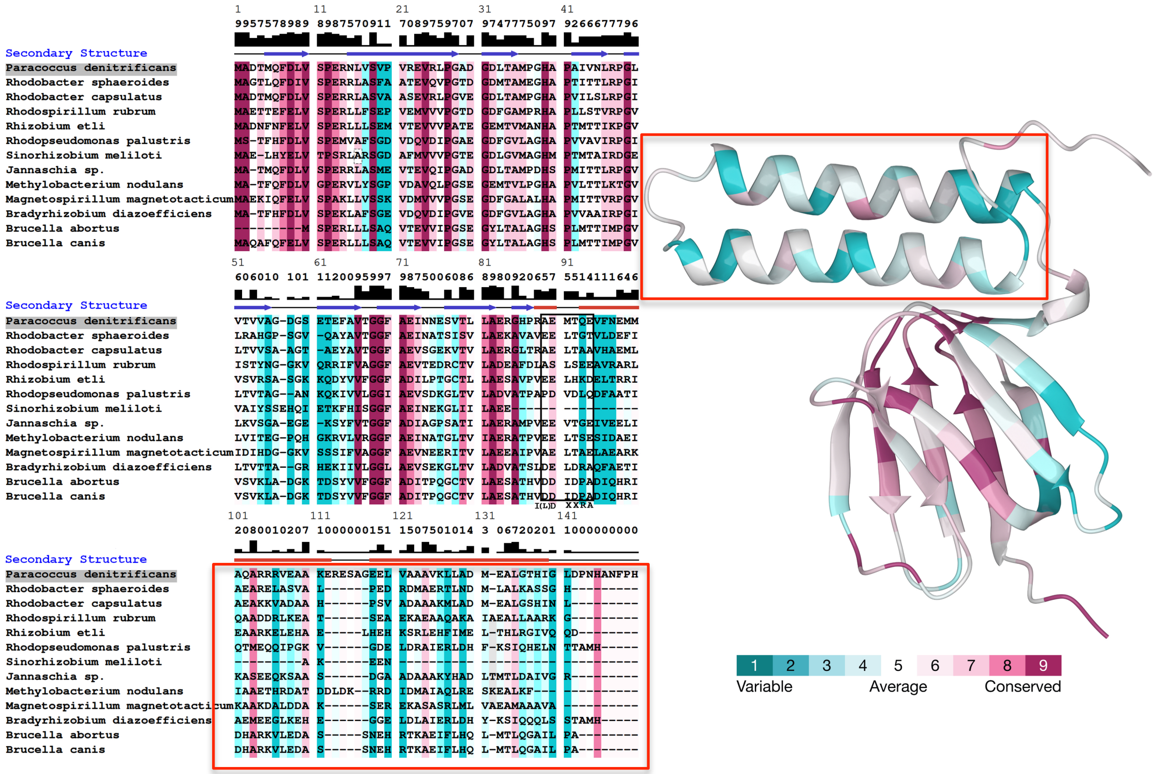
Secondary Structure
Paracoccus denitrificans
Rhodobacter sphaeroides
Rhodobacter capsulatus
Rhodospirillum rubrum
Rhizobium etli
Rhodopseudomonas palustris
Sinorhizobium meliloti
Jannaschia sp.
Methylobacterium nodulans
Magnetospirillum magnetotacticum
Bradyrhizobium diazoefficiens
Brucella abortus
Brucella canis
1 2 3 4 5 6 7 8 9
Variable
Average
Conserved

Figure S1. Conservation of the inhibitory ζ subunit and the non-inhibitory ε subunit of the ATP synthase of α-proteobacteria. A) The α-proteobacterial F1-ATPase inhibitory ζ subunit updated sequences derived from the NCBI were aligned as described in Materials and Methods. The most conserved residues are highlighted in red wine color. The decreasingly less conserved residues are shown in lighter shades of red; the average conserved residues are shown in white; and the increasingly variable or less conserved residues are shown in darker shades of blue (see bottom color-coded conservation scores below the ribbon protein structures). The Pd-ζ structure shown is the most representative average structure of the soluble (NMR) (PDB_id 2LL0), displaying the same color-coded conservation scores of the software Consurf. This updates a previous version of a similar ζ alignment [11]. The most conserved (reddish or cherry-red) region is the N-terminus of ζ which harbors the inhibitory domain of the protein. The conservation decreases towards the C-terminus of α-helix-4 (blue shades). B) A similar alignment of the protein sequences of the α-proteobacterial ε-subunit was carried out for the same α-proteobacterial species, and the same color-coded conservation scores are displayed in primary and tertiary ε structures, with the latter corresponding to the ε subunit of *P. denitrificans* as derived from the Alpha-fold database. As in the case of ζ, the most conserved (reddish) region of the α-proteobacterial ε is the N-terminus, which is the key functional region of the globular β-sheet domain of ε, whose function is to connect the γ subunit from the rotor and F_1, to the *c* subunit of the rotor and F_O. The less conserved region of the α-proteobacterial ε is the C-terminal α-helix hairpin, which has lost its inhibitory and ATP binding properties in *P. denitrificans* [6; 7; 27]. The position of the missing ATP binding cassette (I(L)DXXRA) observed in other inhibitory ε subunits from non-α-proteobacteria is in a black box showing that this ATP binding motif is lost in most, if not all ATP synthases´ ε subunits from α-proteobacteria (see Figure S1.1). Red boxes indicate the inhibitory N-terminus of ζ, and the inhibitory C-terminus of the ε subunit from non-α-proteobacteria, which is variable and non-inhibitory in α-proteobacteria.

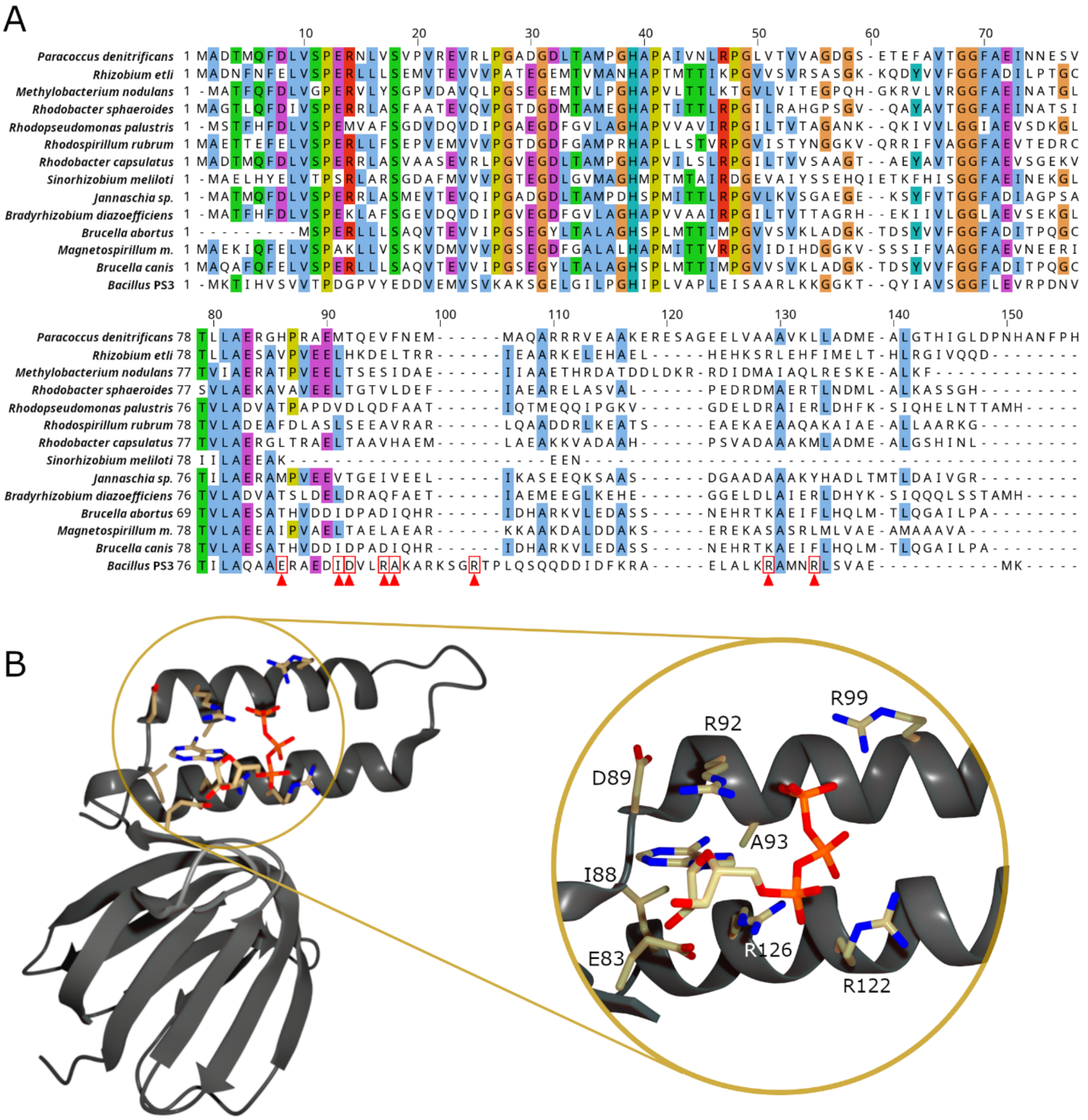

Figure S1.1: Close up to the ATP binding resiudes in the ε subunit from non-α-proteobacteria. A) Multiple sequence alignment of the ε subunit of multiple α-proteobacteria and *Bacillus* PS3. For the *Bacillus* PS3 sequence, the residues involving in ATP binding are highlighted in red. **B)** Crystal structure of the ε subunit and ATP complex from *Bacillus* PS3 (PDB ID 2E5Y). Only the residues involved in ATP binding, and ATP itself, are colored. A close up of the ATP binding site is shown. It is highlighted that the ε-ATP binding motif and residues are in general lost in the ε subunits of the F_1F_O-ATP synthase of *Paracoccus denitrificans* and related α-proteobacteria (Figure S1B, black box).

Figure S2

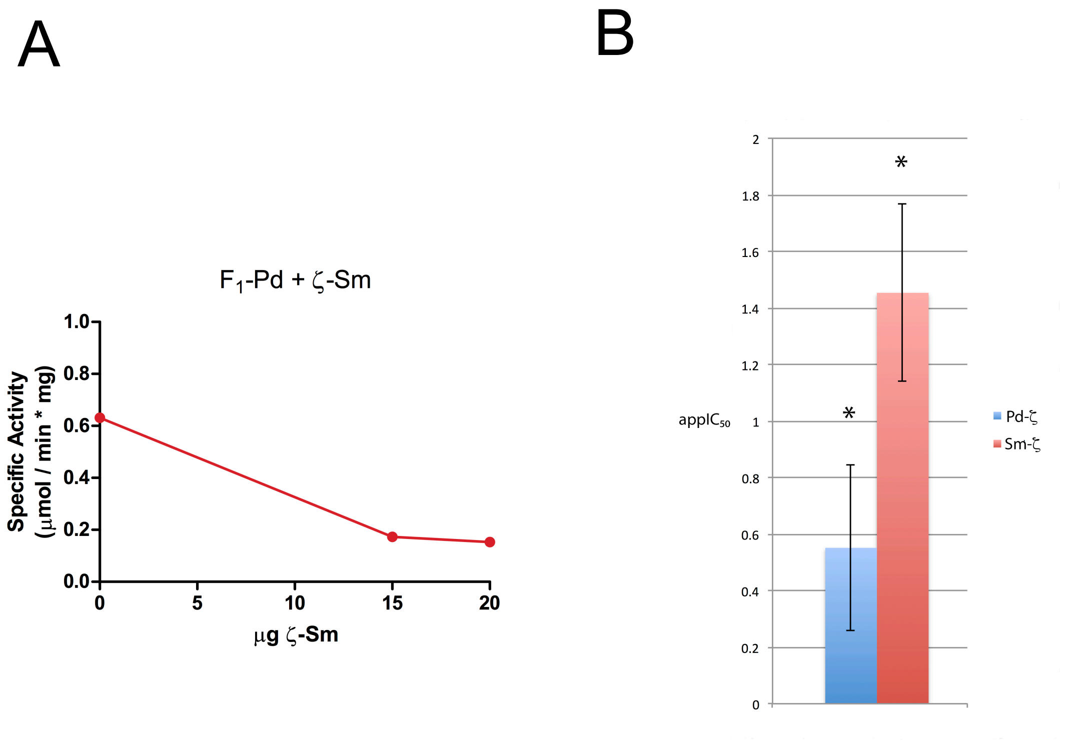

Figure S2. Reconstitution and inhibition of Sm-ζ on the PdF_1-ATPase and the PdF_1F_O-ATPase of SBP. A) Heterologous reconstitution of SmF_1 with increasing amounts of recombinant Pd-ζ. Reconstitution and PdF_1-ATPase assays were carried out as described in Materials and Methods, mixing the indicated amounts of Sm-ζ subunit with 6.5 µg of PdF_1WT previously. The PdF_1-ATPase activity is relatively low due to the presence of the endogenous ζ subunit. B) To estimate an IC50 for Sm-ζ and Pd-ζ subunits for the PdATP synthase, we used the $PdF_1F_O{}^{\Delta\zeta}$-ATPase of SPB derived from the $PD^{\Delta\zeta}$ mutant ($SBP^{Pd\Delta\zeta}$), lacking the endogenous ζ subunit completely. Separate experiments and curve fittings were carried out in triplicate for Pd-ζ and Sm-ζ in $SBP^{Pd\Delta\zeta}$ (figures 6B and 6C, respectively). The derived ${}_{app}IC_{50}$ values from curve fittings were averaged for Pd-ζ = 0.55±0.36 µM (±SD) and for Sm-ζ = 1.45±0.38 µM and plotted in bars (blue, Pd-ζ, red Sm-ζ). A Student's t-test showed the significance (*) in the difference of a 2.6-fold increase in IC50 of Sm-ζ in relation to Pd-ζ ($p<0.005$)

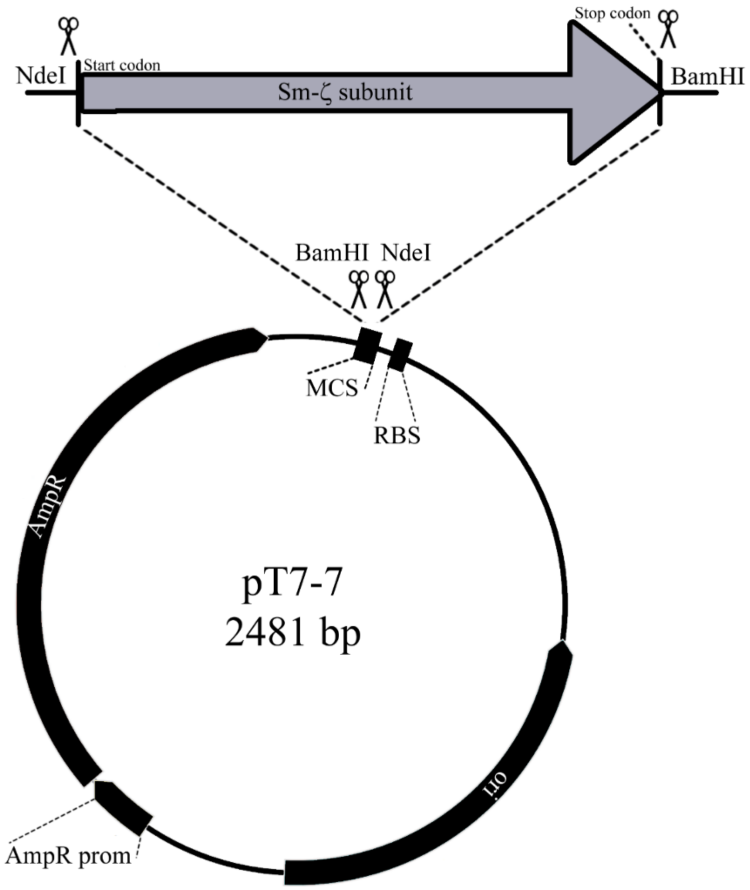

Figure S3. **Recombinant *Sinorhizobium meliloti* ζ construct**. Depiction of the Sm-ζ sequence and its ligation into the pT7-7 plasmid. The Sm-ζ sequence was amplified by using specific primers flanked by restriction enzyme recognition sequences (shown as NdeI and BamHI). The scissors show where the digestion enzymes cut and then where the complementary digested sequences were ligated. The start codon was flanked by the NdeI sequence, making sure that the start codon was in the correct reading frame after the ribosome binding sequence (RBS) sequence.

Supplementary Table 1. Primers and sequence amplified

**Primers**	**Sequence**	**Characteristics**

<table>
<tr><td colspan="3">For Rhizobium etli</td></tr>
<tr><td>FORWARD</td><td>5’- CAT ATG GGC ATC ATC GAC GAG CGT GAA GAA G -3'</td><td>NdeI-Start codon</td></tr>
<tr><td>REVERSE</td><td>5'- GGA TCC TTA GCC GGC CTC GTC AAG -3'</td><td>BamHI-stop codon</td></tr>
<tr><td colspan="3">Sequence amplified</td></tr>
<tr><td colspan="3">ATGGGCATCATCGACGAGCGTGAAGAAGCACTCGAGAAAGAGTACATCGTGAAGCTCGAGCG
TTCGTTCCATATCAAGGCGAGGCGTGACCGGCTGCTGGCACGCTGGGCGGCCGAGCTCATCG
GAAGAACCGACTTCGACGTCTATTTCGACGAAGTCATTTCCGCCGAGTTGCTGGAGTCAGGAG
ATGAGAACGTCTTCAGAAAGATTCTCAGCGATCTGCAATATGCCGACGTCGCGATCAGCGCTCA
GACGGTTCGCGAGAAAATGAAGCAGCTTCTGCTTGAAGCGGCAAGGTCCCTTGACGAGGCCG
GCTAA</td></tr>
<tr><td colspan="3">For Sinorhizobium meliloti</td></tr>
<tr><td>FORWARD</td><td>5’- CAT ATG ACC ACG ATG CAG GAT CGC GAG AAG GC - 3’</td><td>NdeI-Start codon</td></tr>
<tr><td>REVERSE</td><td>5’- GGA TCC TCA GTT TTT CTG TAG CTG TGC GAC CGC TTC - 3’</td><td>BamHI-stop codon</td></tr>
<tr><td colspan="3">Sequence amplified</td></tr>
<tr><td colspan="3">ATGACCACGATGCAGGATCGCGAGAAGGCTTTCGAGGCGAAGTTCGCACTGGACGAGGAGTTGAGG
TTCAAGGCGACCGCACGCCGCAACAAACTGCTTGGCCTTTGGGCCGCCGGTCTGCTCGCCAAGTCG
GACCCGGAAGCCTATGCGAGCGAAATCGTCGCCGCGGACTTCGAAGAGGCCGGGCACGAGGACGTC
GTGCGCAAGATCAAGACCGATTTCGATGCGGCCGGCGTTGCCATATCCGAAGACGACATTCGCGTC
CGCATGATTGAGTTGCTCTCGGAAGCGGTCGCACAGCTACAGAAAAACTGA</td></tr>
</table>

For data of Pd-ζ see reference [39]

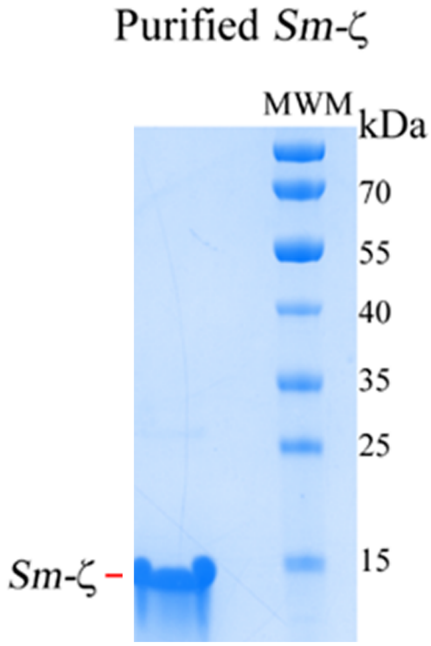

Figure S4. Purification of recombinant ^{15}N, ^{13}C, double-labeled *Sinorhizobium meliloti* ζ-subunit. SDS-PAGE of purified **uniformly double** labeled Sm-ζ. On the right lane is the molecular weight marker (MWM), and on the left is the purified Sm-ζ.

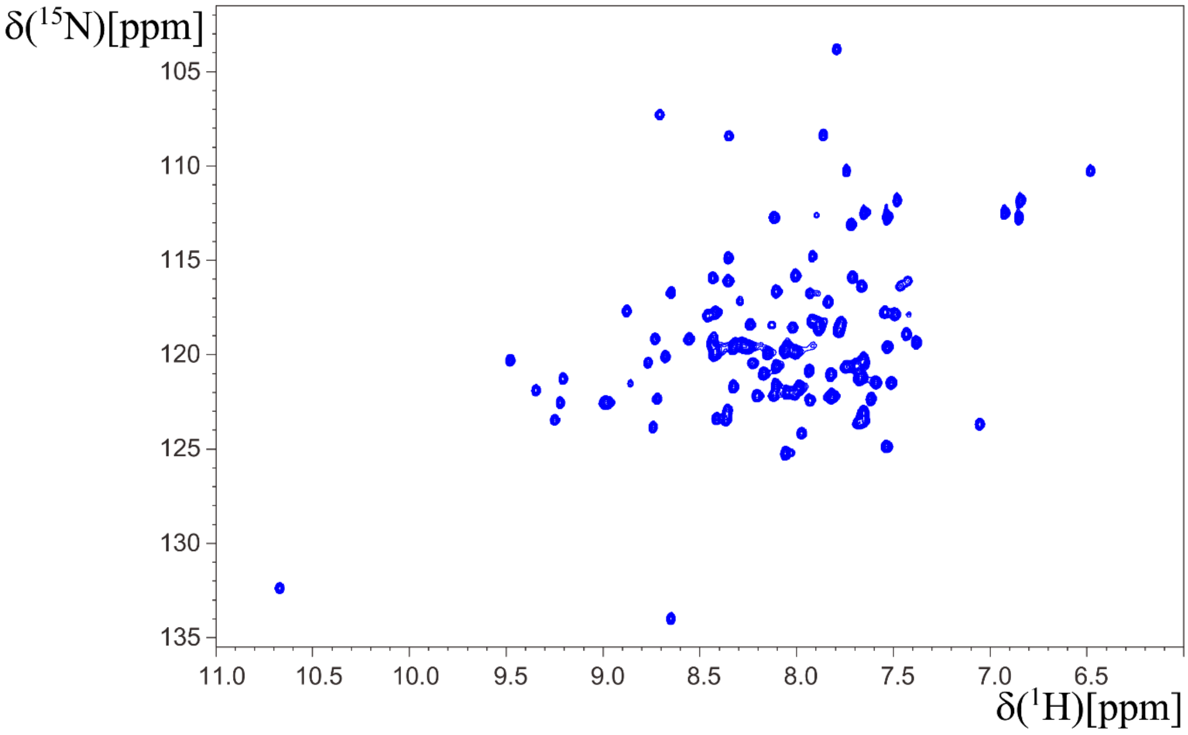

Figure S5. [^{15}N, ^{1}H] HSQC of uniformly double labeled purified ζ subunit from *Sinorhizobium meliloti*. The [^{15}N, ^{1}H] HSQC spectrum shows well-dispersed N-H correlation peaks. On the left axis (δ_1), the nitrogen chemical shifts signals, and on the bottom axis (δ_2), the proton chemical shifts signals.

Supplementary Table 2. Summary of structure quality factors.

Quantity	Value
	ζ-subunit (1-101)
NOE restraints	1021
Intra-residual	295
Short-range	301
Medium-range	285
Long-range	140
Dihedral angle constraints	643
Residual target-function value ($A^{\circ 2}$)	0.76 +/- 0.13
Setup-given RMSD range	1-101
Backbone RMSD (A˚)	1.23+/- 0.19
Heavy atom RMSD (A˚)	1.77+/- 0.17
Optimal RMSD range	22-55, 61-97
Backbone RMSD (A˚)	0.69+/- 0.11
Heavy atom RMSD (A˚)	1.17+/- 0.13
-Most favoured regions (%)	86.06
-Additional allowed regions (%)	13.14
-Generously allowed regions (%)	0.59
-Disallowed regions (%)	0.21
AMBER energies[&]	
Total	-4347 Kcal/mol
van der Waals	-296 Kcal/mol

Electrostatic	-4926 Kcal/mol

The top 17 entries represent the input generated in the final cycle of the cyana by J-unio calculation.

[&] Structure energy minimization calculated by GROMACS.

The structure calculated by J-unio starts from the 4th a.a. (the red M in the sequence)

MTTMQDREKAFEAKFALDEELRFKATARRNKLLGLWAAGLLAKSDPEAYASEIVA
ADFEEAGHEDVVRKIKTDFDAAGVAISEDDIRVRMIELLSEAVAQLQKN

Supplementary Table 3.

<table>
<tr><th>Sequence identifier</th><th>Alpha Fold link</th><th>Supplementary Figure S6 identifier</th><th>Color Coding</th></tr>
<tr><td>A0A220MWR4</td><td>https://alphafold.ebi.ac.uk/entry/A0A220MWR4</td><td>A and B</td><td>Red</td></tr>
<tr><td colspan="4">MTTMQDREKAFEAKFALDEELRFKATARRNKLLGLWAAGLLAKSDPEAYASEIVAADFEEAGHEDVVRKIKTDFDAAGVA
ISEDDIRVRMIELLSEAVAQLQKN</td></tr>
<tr><td colspan="4">ζ_Sm MTTMQDREKAFEAKFALDEELRFKATARRNKLLGLWAAGLLAKSDPEAYASEIVAADFEE 60
1_ζSm_A0A220MWR4 MTTMQDREKAFEAKFALDEELRFKATARRNKLLGLWAAGLLAKSDPEAYASEIVAADFEE 60
****

ζ_Sm AGHEDVVRKIKTDFDAAGVAISEDDIRVRMIELLSEAVAQLQKN 104
1_ζSm_A0A220MWR4 AGHEDVVRKIKTDFDAAGVAISEDDIRVRMIELLSEAVAQLQKN 104
****
100 % identity with ζ_Sm</td></tr>
<tr><td></td><td></td><td></td><td></td></tr>
<tr><td>A0A2J0YZ70</td><td>https://alphafold.ebi.ac.uk/entry/A0A2J0YZ70</td><td>C and D</td><td>Orange</td></tr>
<tr><td colspan="4">MTTMQDREKAFEAKFALDEELRFKATARRNKLLGLWAAGLLAKSDPEAYASEIVAADFEEAGHEDVVRKIKADFDAAGVA
ISEDDIRVRMIELLSEAVAQLQKN</td></tr>
<tr><td colspan="4">ζ_Sm MTTMQDREKAFEAKFALDEELRFKATARRNKLLGLWAAGLLAKSDPEAYASEIVAADFEE 60
2_ ζSm_A0A2J0YZ70 MTTMQDREKAFEAKFALDEELRFKATARRNKLLGLWAAGLLAKSDPEAYASEIVAADFEE 60
****

ζ_Sm AGHEDVVRKIKTDFDAAGVAISEDDIRVRMIELLSEAVAQLQKN 104
2_ζSm_A0A2J0YZ70 AGHEDVVRKIKADFDAAGVAISEDDIRVRMIELLSEAVAQLQKN 104
***********:********************************
99 % identity with ζ_Sm</td></tr>
<tr><td></td><td></td><td></td><td></td></tr>
</table>

A0A8B3MEF7	https://alphafold.ebi.ac.uk/entry/A0A8B3MEF7	E and F	Light green

```
MNNIRDRQQGFEKKFAMDEETKFKAMARRNKLFGLWAAEKLGKAGTDADAYAKEVVQADFEEAGDNDVFRKVRTDFDAAG
VVLSDSQIRSTMDELLAAAAEQIKNS
```

```
ζ_Sm                MTTMQDREKAFEAKFALDEELRFKATARRNKLLGLWAAGLLAKS--DPEAYASEIVAADF   58
3_ζSm_A0A8B3MEF7    MNNIRDRQQGFEKKFAMDEETKFKAMARRNKLFGLWAAEKLGKAGTDADAYAKEVVQADF   60
                    *..::**::.** ***:*** :*** ******:*****  *.*:  * :***.*:* ***

ζ_Sm                EEAGHEDVVRKIKTDFDAAGVAISEDDIRVRMIELLSEAVAQLQKN         104
3_ζSm_A0A8B3MEF7    EEAGDNDVFRKVRTDFDAAGVVLSDSQIRSTMDELLAAAAEQIKNS         106
                    ****.:**.**::********.:*:.:**  * ***: *. *:::.
```

58.65 % identity with ζ_Sm

A0A3S2TBR5	https://alphafold.ebi.ac.uk/entry/A0A3S2TBR5	G and H	Dark green

```
MSIRDRQEGFEKKFAMDEETKFKAMARRNKLFGLWAAEKLGKTGTDADTYAKEVVQADFEEVGDNDVFRKVRTDFDVAGV
VLSDTQIRSIMDELLAAAVEQIKNN
```

```
ζ_Sm                MTTMQDREKAFEAKFALDEELRFKATARRNKLLGLWAAGLLAKS--DPEAYASEIVAADF   58
4_ζSm_A0A3S2TBR5    -MSIRDRQEGFEKKFAMDEETKFKAMARRNKLFGLWAAEKLGKTGTDADTYAKEVVQADF   59
                      :::**::.** ***:*** :*** ******:*****  *.*:  * ::**.*:* ***

ζ_Sm                EEAGHEDVVRKIKTDFDAAGVAISEDDIRVRMIELLSEAVAQLQKN         104
4_ζSm_A0A3S2TBR5    EEVGDNDVFRKVRTDFDVAGVVLSDTQIRSIMDELLAAAVEQIKNN         105
                    **.*.:**.**::****.***.:*: :**  * ***: ** *:::*
```

57.28 % identity with ζ_Sm

A0A843ZLT3	https://alphafold.ebi.ac.uk/entry/A0A843ZLT3	I and J	Dark Blue

MSIRDRQEGFEKKFAMDEETKFKAMARRNKLLGLWAAEKLGKTGTDAVAYAKEVVQADFEEAGDNDVFRKVRTDFDTA

GVVLSDTQIRSIMDELLATAVEQIKNN

```
ζ_Sm               MTTMQDREKAFEAKFALDEELRFKATARRNKLLGLWAAGLLAKS--DPEAYASEIVAADF   58
5_ζSm_A0A843ZLT3   -MSIRDRQEGFEKKFAMDEETKFKAMARRNKLLGLWAAEKLGKTGTDAVAYAKEVVQADF   59
                     :::**::.** ***:*** :*** ************  *.*:  *  ***.*:* ***

ζ_Sm               EEAGHEDVVRKIKTDFDAAGVAISEDDIRVRMIELLSEAVAQLQKN         104
5_ζSm_A0A843ZLT3   EEAGDNDVFRKVRTDFDTAGVVLSDTQIRSIMDELLATAVEQIKNN         105
                   ****.:**.**::****:***.:*: :**  * ***: ** *:::*
```

60.19 % identity with ζ_Sm

A0A7G6QIK6	https://alphafold.ebi.ac.uk/entry/A0A7G6QIK6	K and L	Cyan

MSIRDRQEGFEKKFAMDEETKFKAMARRNKLLGLWAAEKLGKTGTDADAYAKEVVQADFEEAGDNDVFRKVRTDFDAA
GVVLSDTQIRSIMDELLATAVEQIKNH

```
ζ_Sm               MTTMQDREKAFEAKFALDEELRFKATARRNKLLGLWAAGLLAKS--DPEAYASEIVAADF   58
6_ζSm_A0A7G6QIK6   -MSIRDRQEGFEKKFAMDEETKFKAMARRNKLLGLWAAEKLGKTGTDADAYAKEVVQADF   59
                     :::**::.** ***:*** :*** ************  *.*:  * :***.*:* ***

ζ_Sm               EEAGHEDVVRKIKTDFDAAGVAISEDDIRVRMIELLSEAVAQLQKN         104
6_ζSm_A0A7G6QIK6   EEAGDNDVFRKVRTDFDAAGVVLSDTQIRSIMDELLATAVEQIKNH         105
                   ****.:**.**::********.:*: :**  * ***: ** *::::
```

60.19 % identity with ζ_Sm

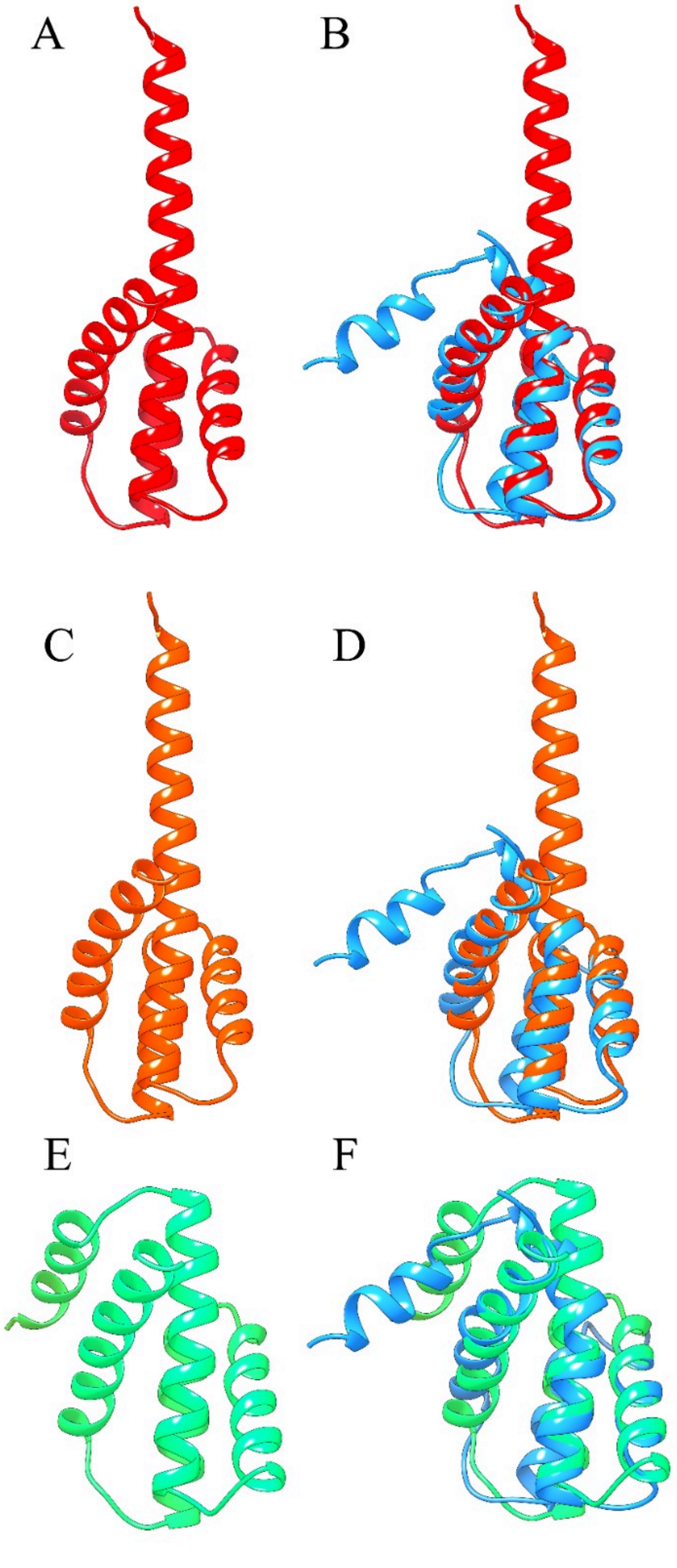
A
B
C
D
E
F

Figure S6. **Alpha fold predicted structures of Sm-ζ.** Alpha fold (AF) predicts six structures of Sm-ζ, and they are compared here. These six structures come from different sequences derived likely from different strains (sequence details in Table S3). When compared to the sequence of our Sm-ζ determined by solution NMR, they have 100 % (red, A), 99 % (orange, C), 58.65 % (light green, E), 57.28 % (dark green, G), 60.19 % (dark blue, I), 60.19 % (cyan, K) identity (identity details in table S3). Each one of these structures was supper imposed with our Sm-ζ structure; our determined structure is in light blue (B, D, F, H, J, and L). The AF models with sequences with ~ 60% identity to our Sm-ζ (PDB_id 7VKV) have similar N-terminus folding (E-L), whereas the AF models with sequences of 100 % and 99 % identity with our NMR structure had an extended N-terminus (A-D).

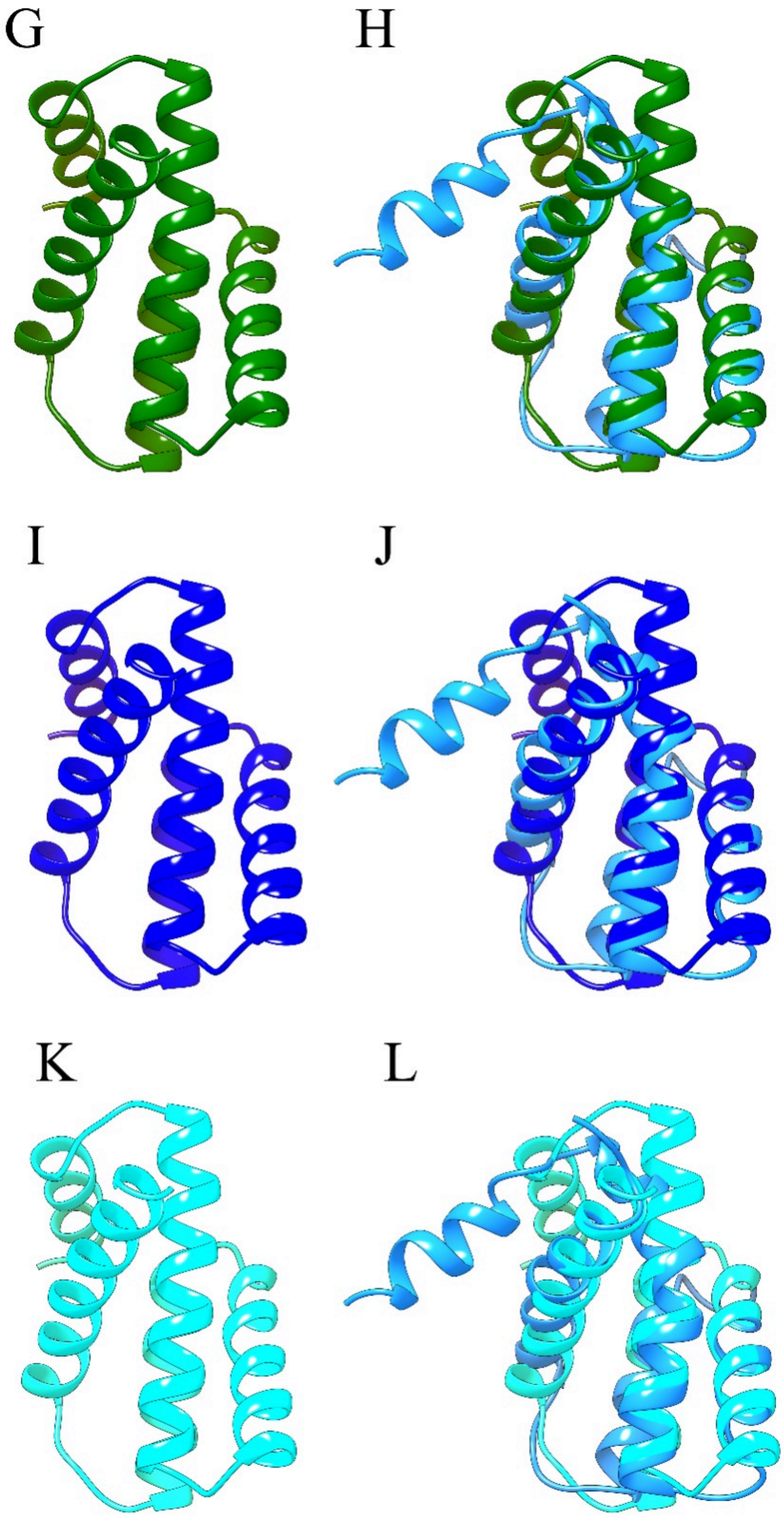

Continuation of Figure S6.

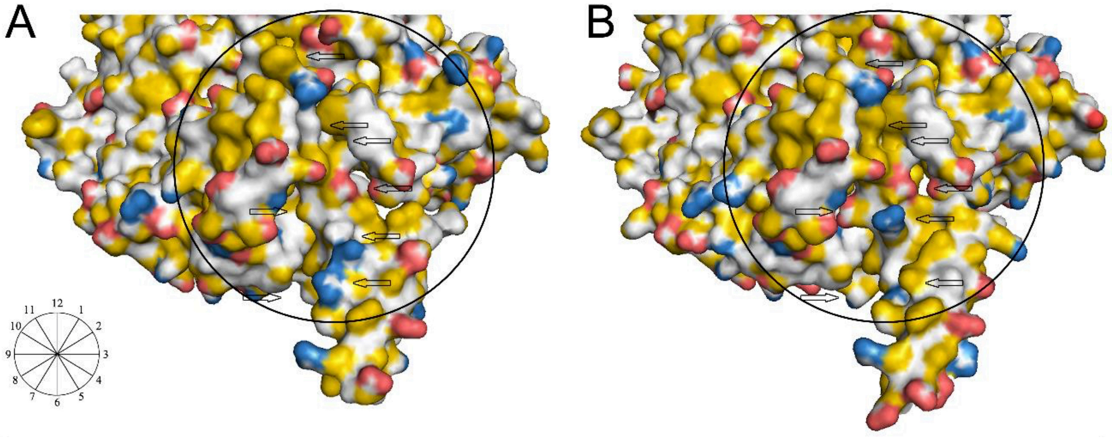

Figure S7. Model of the ζ subunit inhibitory binding site at the α_{DP}/β_{DP} interface of *S. meliloti* SmF_1-ATPase. The binding site of the Sm-ζ subunit at the INGECORE of the SmF_1-ATPase was modeled with the Swiss Model Prot using the subunits of *P. denitrificans* from PDB_id 5DN6 as a template. Subunits are shown on the surface model, and the side chains of amino acid residues were colored using the YRB script: in the surface structure, red color implies negative charge, blue color implies positive charge, yellow implies hydrophobicity, and white implies polarity. A) Shows the α_{DP}, β_{DP} and ζ subunits of *P. denitrificans*. B) Shows the α_{DP}, β_{DP} and ζ subunits of *S. meliloti*. The interaction area with the α_{DP}, β_{DP} subunits is delimited in the circle. The arrows indicate places where there are different changes in the SmF_1-Sm-ζ and PdF_1-Pd-ζ structures. The circle divided into 12 slices serves to identify the regions into which the circle of the interface with the ζ subunit is imaginarily divided. Images were constructed in PyMol. At the interface of ζ with α and β of *S. meliloti*, eight changes are shown in the circle, each marked with an arrow. The change at 12' shows the disappearance of a hydrophobic protuberance. The two changes in the center of the circle show two hydrophobic zones where in PdF_1F_O-ζ there was a polar region and a small cavity. The change at 4' shows a polar region's disappearance and a small gap's appearance. The change at 5' shows the appearance of a positively charged protrusion. The change between 5' and 6' shows the disappearance of a positive charge and the appearance of a hydrophobic region. The change at 7' shows the

appearance of a polar density. The change at 8' shows the appearance of a surface with two negative charges. In summary, in the modeled structure of the subunits of the SmF_1-ATPase (α, β, γ and ζ), there are several clear changes in comparison with the structure PdF_1F_O-Pd-ζ of *P. denitrificans* (PDB_id 5DN6). The structural changes show sites where the modifications may be related to the inability of the ζ subunit of *S. meliloti* to inhibit its F_1-ATPase, and its still inhibitory function on the F_1-ATPase of *P. denitrificans* (Figure 6). In summary, changes in the ζ subunits as well in the INGECORE interface of the F_1-subunits may be responsible for the functional differences between Pd-ζ and Sm-ζ, and PdF_1 and SmF_1. This and more detailed structural comparisons have been presented previously in the Ph.D. thesis of Francisco Mendoza-Hoffmann, to identify in the future some of the key residues putatively responsible for the functional results here observed (Ref. [44]).

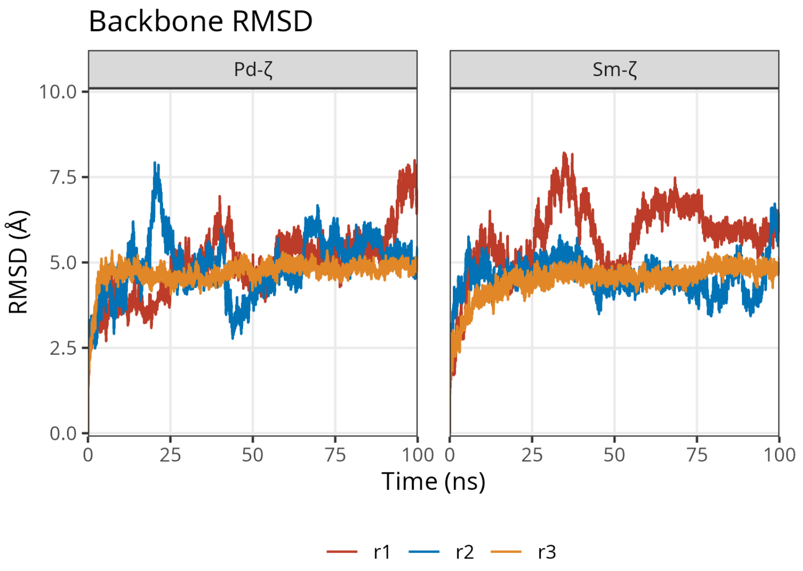

Figure S8A. Figure Root mean square deviation (RMSD) plot of Pd-ζ and Sm-ζ during the 100 ns constant pH simulations at pH 8, calculated using the backbone atoms of each structure. The first (red), second (blue), and third (yellow) replicas are shown for both systems.

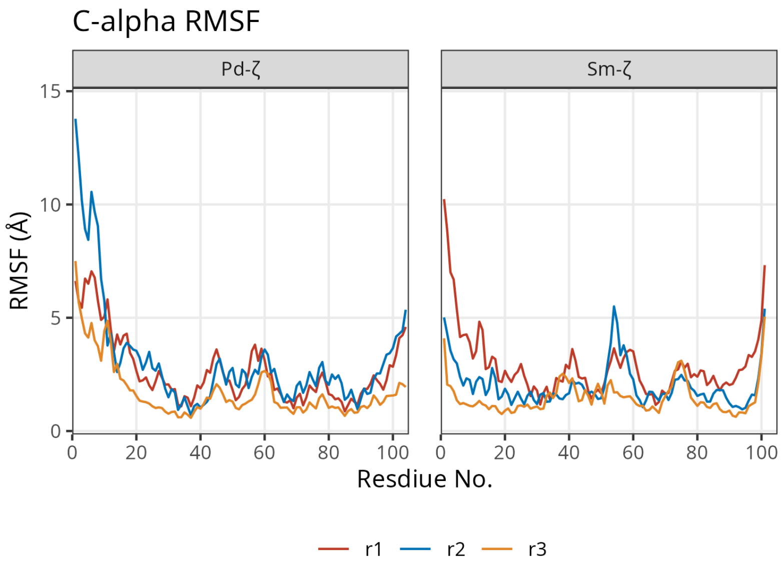

Figure S8B. Root mean square fluctuation (RMSF) plot of the Cα atoms of Pd- ζ and Sm- ζ during the 100 ns constant pH simulations at pH 8. The first (red), second (blue), and third (yellow) replicas are shown for both systems.

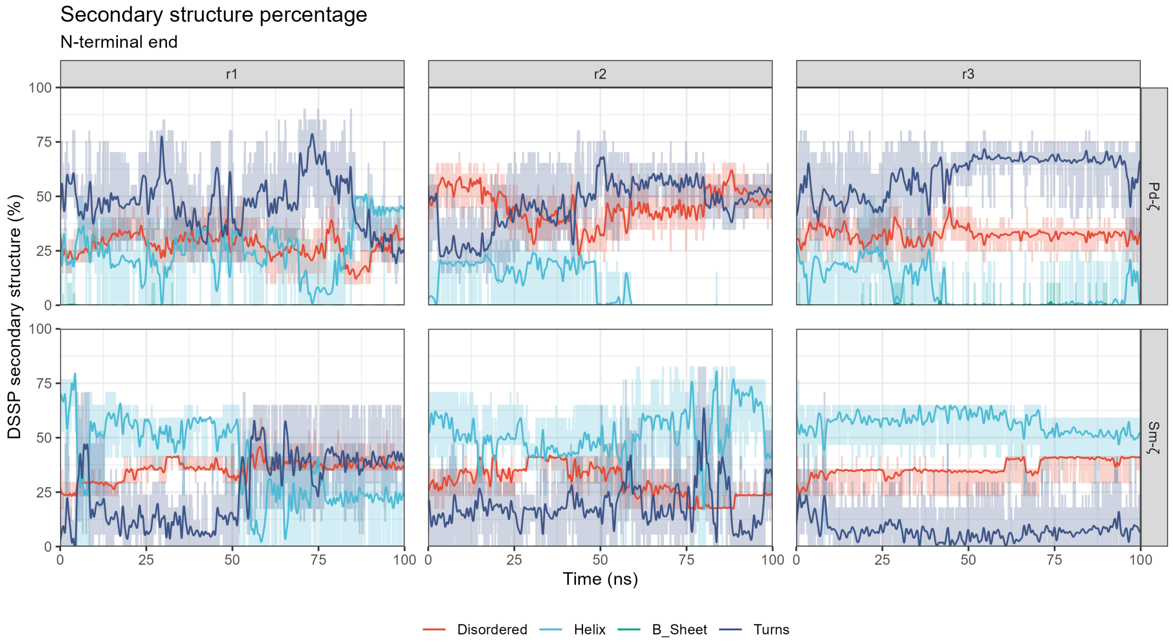
Secondary structure percentage
N-terminal end
r1
r2
r3
Pd-ζ
Sm-ζ
DSSP secondary structure (%)
Time (ns)
Disordered
Helix
B_Sheet
Turns

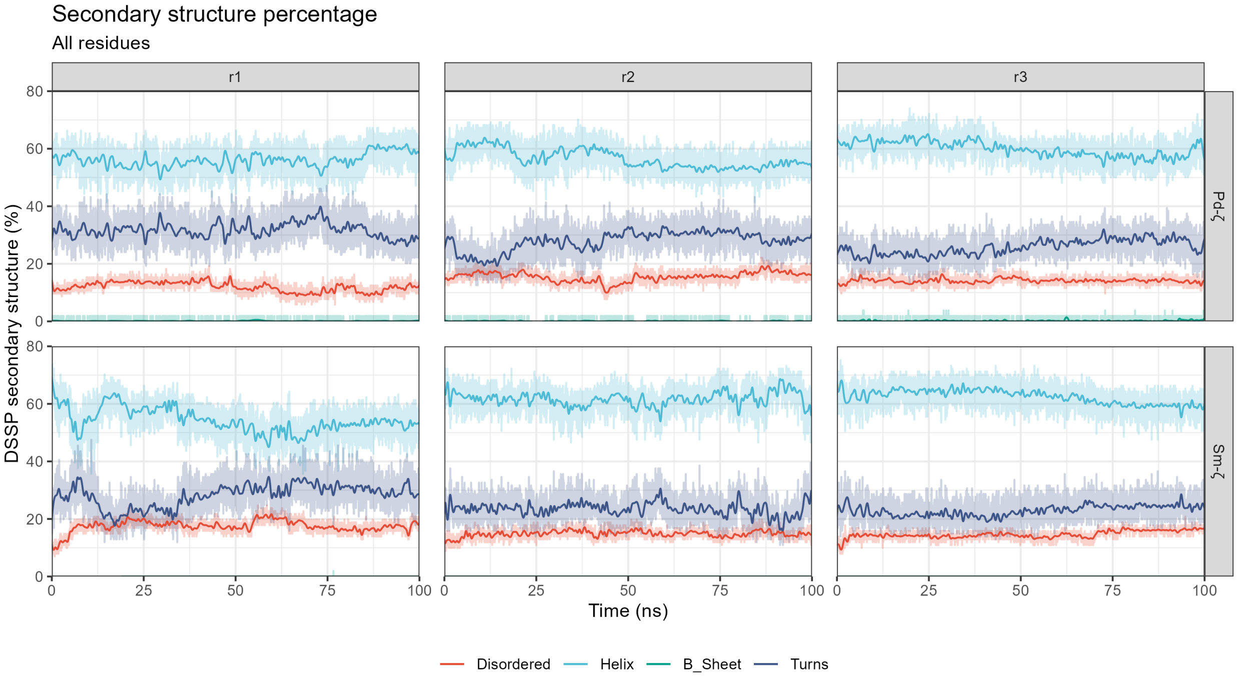
Secondary structure percentage
All residues
r1
r2
r3
Pd-ζ
Sm-ζ
DSSP secondary structure (%)
Time (ns)
Disordered
Helix
B_Sheet
Turns

Figure S8C. Secondary structure percentage computed with the DSSP algorithm for all different replicas of the simulations of Pd-ζ and Sm-ζ at constant pH 8.0. The helix content (light blue) is calculated by adding the α-helix, π-helix and 3_{10}-helix, and the turns (dark blue) by the addition of the turns and bends content. The β-sheets (green), when they occur, correspond to anti-parallel β-sheets. The shadowed lines shows the overall secondary structure percentage for each type, while the solid lines are the smoothing spline for each secondary structure type. The secondary structure composition for the entire structures (top) and the N-terminal end (bottom) are shown. Supplementary Video1 corresponds to MD1 (r1) of Pd-ζ, with the formation of the extended inhibitory α-helix in light blue, and the Supplementary Video 2 corresponds to r2 where the α-helical content of the Sm-ζ N-terminus seems to increase, but it does not reach the extended N-terminal inhibitory conformation oberved for Pd-ζ.

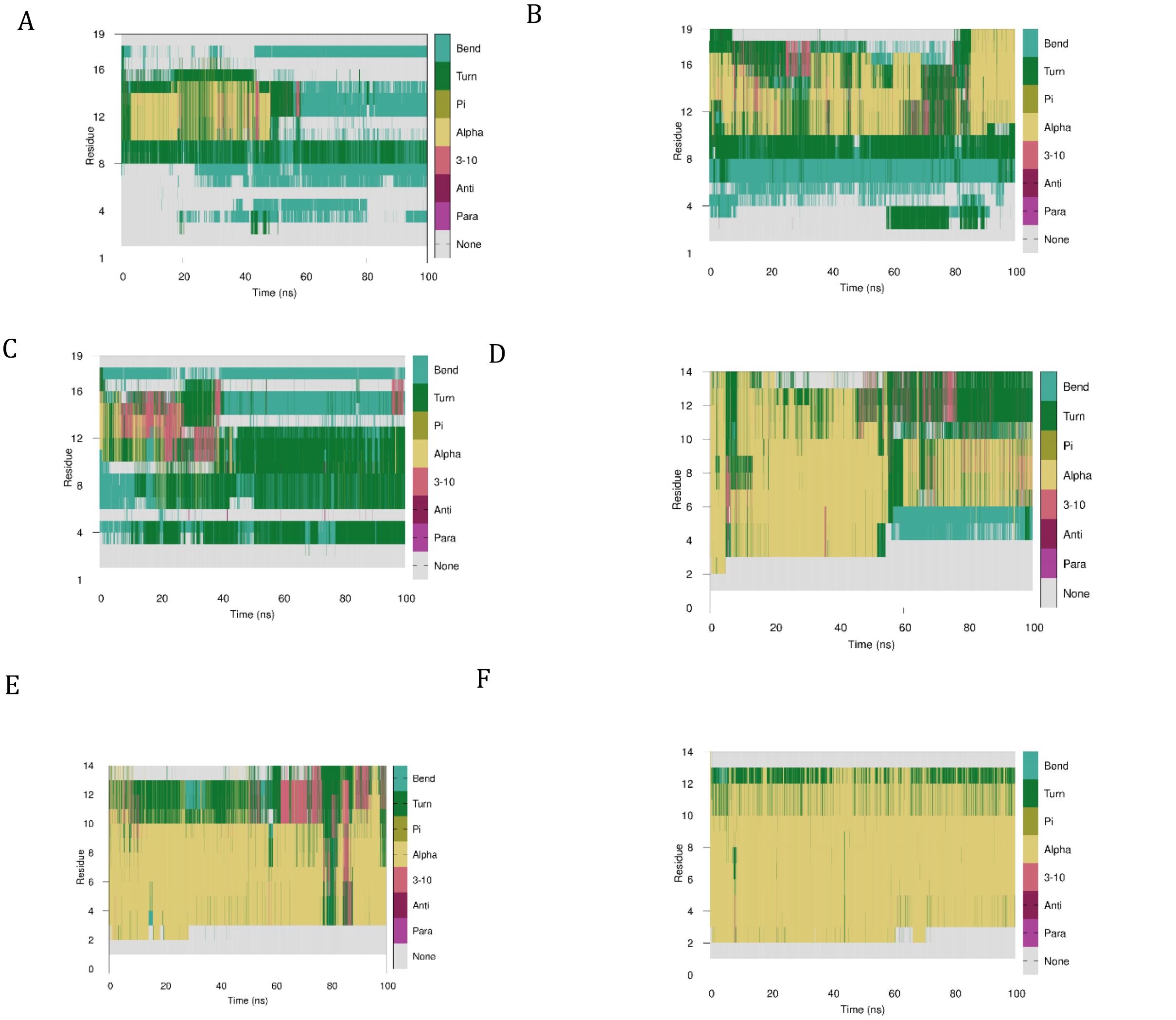

Figure S8D. Per-residue secondary strucutre for Pd-ζ (A-C) and Sm-ζ (D-F) computed using DSSP. Each replica is depicted individually.

Supplementary video 1. MD of the Pd-ζ at constant pH 8.0. The 100 ns MD of Pd-ζ was carried out at constant pH 8.0 as described in Materials and Methods. The trajectories of the protein atoms were collected in a single video showing that at the end of the 100 ns, the intrinsically disordered region (IDPr) of the Pd-ζ N-terminus shifts from the disordered status, to the formation of a short α-helix, similar to the extended and inhibitory N-terminus α-helical conformation of ζ. In the associated figure (Figure 9) the final Pd-ζ conformer achieved by the MD

simulation is well superimposed to the inhibitory N-terminus conformer productively bound to the PdF_1F_O-ATP synthase in the inhibited state (PDB_id 5DN6). See Supplementary Figure 8, and Materials and Methods for further details.

Supplementary video 2. MD of the Sm-ζ at constant pH 8.0. MD of Sm-ζ was carried out at constant pH 8.0 as described in Materials and Methods identically as carried out for Pd-ζ (Suppl. Video 1). Starting structure was that resolved by NMR of Sm-ζ PDB_id 7VKV, main average conformer, before its preparation for MD. See the Supplementary Figure 8, and Materials and Methods for further details.

Cited References here are enlisted in the main text of this paper.
